# Research Progresses on Technologies and Theory of Blanks with Variable Thicknesses

**DOI:** 10.3390/ma17184450

**Published:** 2024-09-10

**Authors:** Xiaogong Wang, Sai Wang, Rihuan Lu, Yanni Xuan, Sijia Zhang, Guangji Zhang, Xianlei Hu, Xianghua Liu, Liansheng Chen

**Affiliations:** 1 College of Metallurgy and Energy, North China University of Science and Technology, Tangshan 063210, China; kyckfk@ncst.edu.cn; 2 Zhejiang Laboratory, Research Institute of Interdisciplinary Innovation, Hangzhou 311121, China; ws@zhejianglab.com; 3 School of Mechanical Engineering, Yanshan University, Qinhuangdao 066004, China; lrh@ysu.edu.cn; 4 School of Energy and Power Engineering, Changsha University of Science and Technology, Changsha 410114, China; xuanyanni@csust.edu.cn; 5 The State Key Laboratory of Rolling & Automation, Northeastern University, Shenyang 110819, China; 15840042289@163.com (S.Z.); huxl@ral.neu.edu.cn (X.H.); liuxh@mail.neu.edu.cn (X.L.); 6 Dong Bao Metal Material Technology Ltd., Shenyang 110103, China; zhanggj_eps@163.com

**Keywords:** dual carbon policy, blanks with variable thicknesses (BVTs), manufacturing technologies, rolling theory

## Abstract

Under the background of dual carbon policy as well as energy conservation, blanks with variable thicknesses (BVTs) which act as structural components have drawn extensive attention due to their excellent strength and formability and reasonable load-bearing distribution characteristics, particularly in the field of automotive manufacturing. With these advantages, the manufacturing technologies of these plates using more efficient rolling methods have thus emerged. This article summarizes four methods and their characteristics for manufacturing plates with variable thicknesses based on rolling technology. In addition, a review is conducted on the latest research progress of the metal flow and rolling theories of existing plates with different thicknesses in the longitudinal and transverse direction.

## 1. Introduction

As the report by the Intergovernmental Panel on Climate Change (IPCC) shows, the impacts of climate change are being witnessed across the world, with increased incidence of events such as extreme heat, floods, and wildfires [1]. Global emissions should be reduced to limit further global warming. According to the roadmap for the global energy sector, technologies for achieving the necessary deep cuts in global emissions by 2030 exist, but staying on the narrow path to net-zero requires their immediate and massive deployment [2]. A major CO_2_ emitter is the transport sector (~15%); in particular, passenger cars are the primary source of CO_2_ emissions [3]. Therefore, reducing the weight of the vehicle body is an effective way to reduce CO_2_ emissions. It is estimated that a 10% reduction in weight vehicle can yield a 6 to 8% reduction in fuel consumption [4].

**Figure 1:**
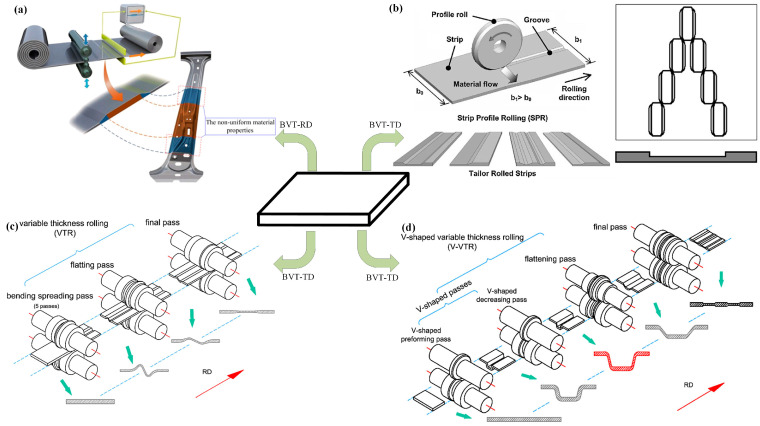
(**a**) Process of VGR [19], (**b**) process of SPR [20], (**c**) process of VTR [21], (**d**) process of V-VTR [21].

The approaches and key technologies for achieving lightweight vehicles mainly include the following aspects: the application of lightweight materials [5,6,7]; lightweight design and optimization of structures [8,9,10]; and the use of new manufacturing process technologies [11,12,13]. Among them, the use of new manufacturing process technologies allows for simplification of the structure, while achieving maximum exploitation of the material’s load-bearing potential during service. Among them, technologies for rolling blanks with variable thicknesses (BVTs) can achieve thickness variation in the sheet or blank along the rolling direction or transverse direction, thus obtaining an optimized load distribution. Blanks with variable thicknesses in the rolling direction (BVTs-RD) are obtained using Variable Gauge Rolling (VGR) or flexible rolling, with the main products including Longitude Profile Plates (LP plates) and Tailor Rolled Blanks (TRBs) [14,15]. Meanwhile, blanks with variable thicknesses in the transverse direction (BVTs-TD) can be obtained using methods such as Strip Profile Rolling (SPR) and Variable Thickness Rolling (VTR), a combined process of Bending–Spreading–Rolling (BSR) and Flattening–Rolling (FR), with the main products including Tailor Rolled Strips (TRSs) and Rolled Profile Strips (RPSs) [16,17]. Research on the theory and technology of BVTs determines the current situation and the future of lightweight vehicles. The processes of the technologies are shown in Figure 1.

It is worth mentioning that TRBs, which are known for their variable thicknesses in the rolling direction, have been widely applied in automotive body manufacturing, including by Volkswagen, Bavarian Motor Works (BMW), and Mercedes Benz [18]. This indicates that BVT can be widely used in automotive lightweighting.

This article introduces the latest overview of four technologies for manufacturing BVTs, of which two are BVTs-RD and the others are BVTs-TD, using the rolling method. In addition, the research on the rolling theories of these technologies is summarized, which includes the biting angle and biting conditions, contact arc formula, forward slip formula, stress equilibrium equation, rolling force formula, etc.

## 2. Technologies and Theories of BVT-RD

### 2.1. Technologies of BVT-RD

**Figure 2:**
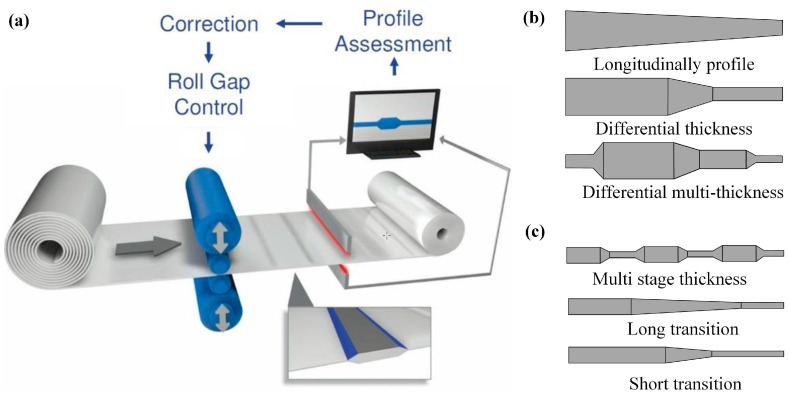
(**a**) Process of TRBs [27], (**b**) several typical sections of LP plates, (**c**) several typical sections of TRBs.

The core technology for manufacturing LP plates and variable thickness plates is essentially flexible rolling [22,23]. LP plates, like regular steel plates, are produced using medium plate rolling mills, with the same production process. The difference lies in the fact that LP plates require continuous changes in the roll gap shape under loaded conditions in the rolling mill, based on the thickness change along the length of the finished steel plate in the rolling passes. This technology can be used in shipbuilding, bridge manufacturing, and construction [24]. TRBs produced by cold rolling, which is also achieved by the dynamic control of the roll gap, can partially be a substitute for Tailor Welded Blanks (TWBs), achieving a lightweight of vehicles [25]. By far, there are a number of successful applications of VGR in the automotive industry, such as in the cross beam, sillboard, connecting beam, stiffener, etc. [26]. By reasonably matching horizontal rolling speed and vertical downward velocity, the longitudinal profiles and dimensions of the TRB and LP plate can be adjusted according to the actual demands. The process of TRBs and the typical products of TRBs and LP plates are shown in Figure 2. The main differences between LPs and TRBs are as follows: (1) different rolling types—an LP plate is hot-rolled while TRBs are cold-rolled; (2) different thicknesses—the thickness of LP plates is usually more than 10 mm, and that of TRBs is generally less than 3 mm; (3) applicable to different working conditions—LP plates can be used for bridges and shipbuilding, while TRBs are often used in automobile manufacturing to reduce the weight of the body.

### 2.2. Forming Theory of BVT-RD

#### 2.2.1. The Biting Angle and the Length of Contact Arc

**Figure 3:**
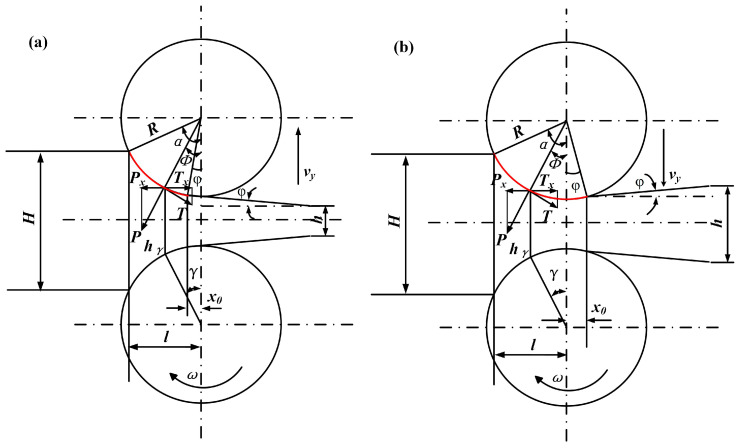
Rolling process of VGR [30], (**a**) UR, (**b**) DR.

Since dynamic adjustment of the roll gap is required during VGR, the forming theory of BVTs-RD is quite different from that of common rolling. Various complex shapes of LP plates and TRB plates can all be considered as a combination of downwards rolling (DR), upwards rolling (UR), and common rolling (CR) over one or multiple stages [28]. In comparison to the conventional rolling process, the rolling process of the wedge section on the longitudinal variable cross-section leads to changes in the exit position of the workpiece, as shown in Figure 3. *T* refers to friction force, *P* to positive pressure, and α to the biting angle. Ignoring the elastic spring-back deformation of the workpiece at the exit and the elastic flattening of the rolls, the transition area rolling has the following characteristics. The line connecting the exit position with the center of the roll must be perpendicular to the inclined surface of the wedge section. The angle between the line connecting the point where the exit position contacts the roll with the center of the roll and the center line of the roll is equal to the inclination angle *φ* of the wedge section. The inclination angle *φ* of each wedge section is constant, so the exit position of the workpiece on the roll remains unchanged. With the rolling of the wedge section, the forward slip value and the exit speed of the workpiece continuously change [29].

The initial bite-in process of VGR is the same as simple rolling. Du [29] believes that *α* in both upward rolling and downward rolling is consistent with that in conventional rolling, meaning that the bite angle α remains constant during the rolling process. As the rolling process continues, the angle *Φ* between the center line of the roll and the resultant force will continuously change. For upwards rolling, *Φ* decreases gradually from the initial *α*. Once the metal fills the rolls, *Φ* becomes (*α* + *φ*)/2. For downwards rolling, *Φ* decreases gradually from the initial *α*. Once the metal fills the rolls, *Φ* becomes (*α* − *φ*)/2. Zhang [30] also pointed out that the friction conditions required for the bite-in of upwards rolling are the highest. For downwards rolling, when the inclination angle is too large, there may be situations where the bite-in can occur smoothly while the rolling process cannot. Dong [31] believes that the bite angle in variable thickness rolling differs from that in conventional rolling. It is not only related to the reduction ∆*h* and the roll radius R but also to the inclination angle *φ* of the wedge section. Sun [32] established a functional relationship between *α* and the vertical movement speed of the roll and time. This expression offers improved accuracy, but it increases the difficulty of solving some rolling parameters involving *α* because time is used as a variable. Considering the calculation accuracy of the bite angle, the latter two expressions are more accurate. However, from the perspective of engineering applications and subsequent dynamic setting of the rolls, the research results of the former two are more convenient to apply. Specific bite-in conditions and continuous rolling conditions are detailed in Table 1.

The deviation of the exit point in VGR leads to a corresponding change in the contact arc length. Based on the geometric characteristics of the deformation zone in VGR, as shown in Figure 3, the expressions for the contact arc length in UR and DR can be easily derived [29,30,33]:

(1) $$l_{up}=\sqrt{R^{2}-{\left(Rcos\alpha_{0}-∆h/2\right)}^{2}}-x_{0},$$

(2) $$l_{down}=\sqrt{R^{2}-{\left(Rcos\alpha_{0}-∆h/2\right)}^{2}}+x_{0},$$

where *l_up_* and *l_down_* are the contact arc length for UR and DR, respectively; *R* is the roller diameter; *α*_0_ is the wedge angle of the workpiece; and ∆*h* is the reduction. *x*_0_ is the distance from the exit to the centerline of the roll. Dong [31] believes that ∆*h*/2 in the above formula is a relatively small quantity that can be neglected, thus obtaining an expression for a contact arc length that is more conducive to calculation. Generally speaking, the reduction is relatively important in the calculation of rolling parameters such as the rolling force, and its value has a significant impact on the calculation results. Given that, neglecting the reduction is not an appropriate simplification method, although it may provide convenience in engineering applications and further dynamic control of rolls.

#### 2.2.2. The Forward Slip Formula

The presence of an inclination angle in the longitudinal VGR process changes the action point of the workpiece leaving the rolls. The conventional formula for forward slip cannot meet the requirements of the longitudinal variable section rolling process. However, the definition formula *f* = (*v_φ_* − *v*_0_)/*v*_0_ still holds, in which *v*_0_ represents the linear velocity and *v_φ_* represents the horizontal exit velocity of the workpiece. The metal flow velocity of the section in the deformation zone which corresponds to the neutral angle is *v_γ_* = *v*_0_cos*_γ_*. According to the constant mass flow principle, *v_γ_h_γ_* = *v_φ_h_φ_*, *h_γ_* can be deduced, where *h_γ_* is the thickness of the workpiece at the neutral plane and *h_φ_* is the thickness of the workpiece at the exit [28,29,30,34]. For the UR process shown in Figure 3a, the geometric relationship *h_γ_* − *h_φ_* = *D*(cos*φ* − cos*γ*) holds. Based on the above conditions, the formula for forward slip during UR can be derived [28]:

(3) $$f_{up}=\frac{D\left(\cos\;\varphi -\cos\;\gamma \right)\cos\;\gamma }{h_{\varphi }}+\cos\;\gamma -1,$$

The formula above also applies to the calculation of forward slip during DR. With *h_φ_* and *γ* in downwards rolling being substituted in, the forward slip value can be obtained. This method is derived based on the definition of forward slip, combined with the geometric relationship between the neutral plane thickness and the exit section thickness in the deformation zone. Using this forward slip calculation method, assuming the entire contact surface is fully sliding and follows Coulomb’s friction law in the VGR process, and based on the conditions of equal front and back tension, as well as equal unit pressure at the neutral plane, Yu [35] derived the neutral plane thickness.

(4) $$h_{\gamma }=h_{\varphi }{\left[\frac{\sqrt{H-h_{\varphi }}+\sqrt{H-h_{\varphi }+\left(2D\mu^{2}-H+h_{\varphi }\right){\left(\frac{H}{h_{\varphi }}\right)}^{\mu \sqrt{\frac{H-h_{\varphi }}{2D}}}}}{\mu \sqrt{2D}+\sqrt{H-h_{\varphi }}}\right]}^{\mu \sqrt{\frac{H-h_{\varphi }}{2D}}},$$

where *h* is the thickness of the workpiece when entering the roll gap and *μ* is the friction coefficient of the rolling process. In order not to solve *γ*, *h_γ_* − *h_φ_ = d*(cos*φ* − cos*γ*) is substituted into Equation (3) to yield the relationship between the forward slip value and neutral plane thickness.

(5) $$f=\frac{\left(D\cos\;\varphi -h_{\gamma }\right)\left(h_{\gamma }-h_{\varphi }\right)}{{Dh}_{\varphi }\cos\;\varphi },$$

Equation (4) can be substituted into Equation (5) to calculate the forward slip value. 

Another idea is that the metal flowing out of the exit section consists of two parts: the metal flowing into the entry section and the metal flowing from the deformation zone due to the vertical movement of the rolls. Based on this mathematical relationship, using the average velocity and thickness of the metal at the neutral plane to express the velocity of the metal at the exit section, and substituting the roll linear velocity into the expression for the forward slip coefficient, another expression for the forward slip value can be derived [31]:

(6) $$f=\frac{R\left(\cos\;\varphi -\cos\;\gamma \right)v_{0}+\lambda v_{y}R\left(\gamma +\lambda \varphi \right)}{h_{\varphi }v_{0}},$$

where *λ* = −1 is for UR, *λ* = +1 is for DR, and *v_y_* is the vertical velocity of the roll.

The first method for calculating forward slip has been applied to industrial production and its accuracy and effectiveness have been verified through actual on-site production. The second calculation method is an improved algorithm based on the first one, with the advantage of avoiding complex solutions for the neutral angle and instead representing the forward slip value using the neutral plane thickness. However, while the neutral plane thickness is easy to solve, its representation is relatively cumbersome, making it inconvenient for industrial applications. Different from the first two methods, the third method uses velocity as an important parameter to represent the forward slip value. This results in an increased number of parameters, but the significant advantage is that the parameters in the forward slip expression are all simple ones that can be obtained without complex solutions. Although this method has not been applied in industrial production, the author believes that it may receive attention in the future due to its ease of application.

#### 2.2.3. The Force Equilibrium Differential Equations

**Figure 4:**
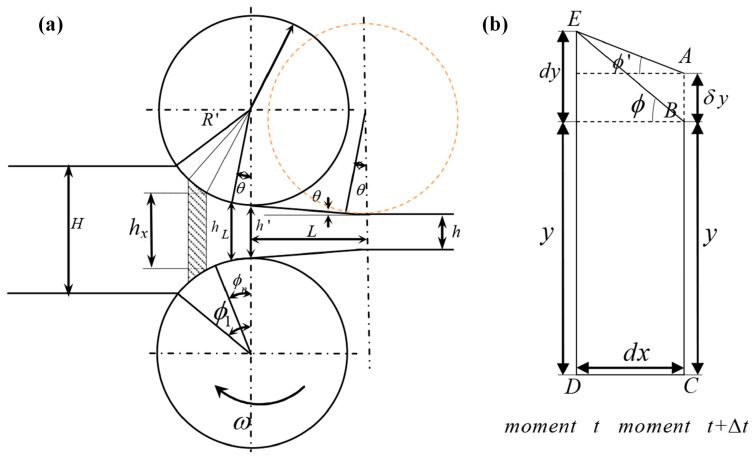
A schematic diagram of an element in the deforming zone during upwards rolling [30]. (**a**) Geometrical relationship of upwards rolling; (**b**) half element in deforming zone.

One important aspect of studying Variable Thickness Rolling is statics analysis. A new force equilibrium equation different from the conventional rolling process was established to derive the calculation formula for rolling force applicable to VGR and provide guidance for the practice of VGR. In the analysis of the VGR process, two assumptions are specifically proposed in addition to the assumption adopted when deriving the Karman Equation [36]. One is that the transition area curve of the workpiece is uniformly continuous, and the other is that the rigid displacement speed of the roll uniformly and continuously changes. Here, the rigid displacement speed refers to the motion speed perpendicular to the rolling direction at the center of roll rotation [23,30,37,38,39]. The unified approach in the literature is to utilize the symmetry in the vertical direction, and half of the elemental with a width of *dx* in the deformation zone is analyzed, as shown in Figure 4.

**Figure 5:**
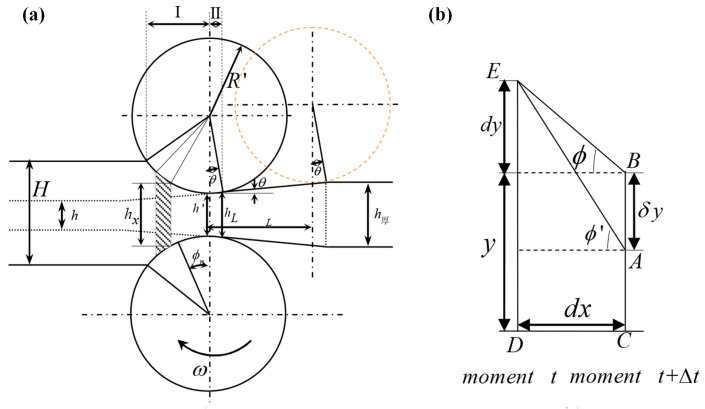
A schematic diagram of an element in the deforming zone during downwards rolling [30]. (**a**) Geometrical relationship of DR; (**b**) half element in deforming zone.

Like conventional rolling, this method is based on the Karman equilibrium differential equation. It is reasonable to take the contact arc between the rolled piece and the roll as a straight line. However, there is also a hidden condition—the shape of the transition zone is assumed to be the straight line type, and also the simplest one. If the curve type changes, the equilibrium differential equation established by this method will no longer be accurate. During upwards rolling, the roll is lifted a distance of *δy* in an interval of ∆*t*, causing the line unit ED to become AC at time *t* + ∆*t*. A thickness of 2(*y* + *δy*) for the workpiece is obtained. From the geometric relationship in Figure 5, it can be inferred that [37]

(7) $$tan\phi =\frac{dy}{dx},$$

(8) $$tan\phi^{\prime }=\frac{dy-\delta y}{dx}=tan\phi -\frac{\delta y}{dx},$$

where *ϕ* is the wedge angle during conventional rolling and *ϕ*′ is that during upwards rolling. Assuming the lifting velocity of the roll during upwards rolling is *v_y_*, and the horizontal velocity is *v_x_*, then it follows that

(9) $$\frac{\delta y}{dx}=\frac{v_{y}}{v_{x}},$$

Substituting Equations (7) and (9) into Equation (8) yields

(10) $$tan\phi^{\prime }=tan\phi -\frac{v_{y}}{v_{x}},$$

A unit width of strip steel was measured to be studied. On the AC line of the elemental body, there is a horizontal tensile stress *σ_x_*, with a resultant force of $f_{ACx}=-\sigma_{x}\left(y+\delta y\right)$. On the ED line, there is a horizontal tensile stress *σ_x_* + d*σ_x_*, with a resultant force of $f_{EDx}=\left(\sigma_{x}+d\sigma_{x}\right)\left(y+dy\right)$.

At the contact surface, $f_{EAb}=p\frac{dx}{cos\;\phi^{\prime }}\sin\;\phi^{\prime }-\mu p\frac{dx}{\cos\;\phi^{\prime }}\cos\;\phi^{\prime }$ is valid in the backward slip zone, and $f_{EAf}=p\frac{dx}{\cos\;\phi^{\prime }}\sin\;\phi^{\prime }+\mu p\frac{dx}{\cos\;\phi^{\prime }}\cos\;\phi^{\prime }$ is valid in the forward slip zone.

According to the force equilibrium condition, the resultant force in the horizontal direction acting on the elemental body is [37]

(11) $$f_{x}=f_{ACx}+f_{EDx}+f_{EAf}=0\;\left(for\;fowward\;slip\;zone\right),$$

(12) $$f_{x}=f_{ACx}+f_{EDx}+f_{EAb}=0\;\left(for\;backward\;slip\;zone\right),$$

The above formulas are rearranged and simplified to

(13) $$y\frac{{d\sigma }_{x}}{dx}+\sigma_{x}tan\phi^{\prime }+p\left(tan\phi^{\prime }\pm \mu \right)=0,$$

where + indicates that the element is located in the forward slip zone, and − indicates that the element is located in the backward slip zone. It can be seen that during the upward rolling, the force balance equation and Karman’s equation are essentially the same. With only the changes in the micro-element shape, the equation can be obtained according to the new geometric relations.

The force equilibrium differential equations can be established in downwards rolling if a similar analysis method as upwards rolling is employed. The difference is that during downwards rolling, the workpiece will deviate towards the exit side when it leaves the contact point with the rolls, resulting in a deformation zone slightly larger than that of the simple rolling process, as shown in Figure 5. The contact arc tangent in the deformation zone of UR changes in the opposite slope, resulting in the non-existence of an equilibrium equation applicable to the whole deformation zone. In the analysis, it is necessary to divide the deformation zone into Zone I and Zone II along the centerline of the two rolls for separate study.

For the elements in Zone I, using the force equilibrium condition, the same force equilibrium equation as in upwards rolling can be obtained, with the only difference being that the size of $\phi^{\prime }$ is not equal. For downwards rolling, $tan\phi^{\prime }=tan\phi +v_{y}/v_{x}$. For the elements in Zone II, the contact arc length from the centerline of the two rolls to the exit can be approximated as ∆*l* = *Rθ*. It can be noted that the elements in Zone II are usually in the forward slip zone. Using the force equilibrium condition, we can obtain [39]

(14) $$d\sigma_{x}+\frac{2\mu p}{h^{\prime }}dx=0,$$

By establishing and solving the force equilibrium equations, the distribution of unit pressure in the deformation zone can be obtained. The formula for calculating the rolling force can then be derived. Zhang [39] established the equilibrium equation for the micro element in the deformation zone in the vertical direction:

(15) $$\sigma_{y}dx\pm \mu p\frac{dx}{\cos\;\phi^{\prime }}\sin\;\phi^{\prime }+p\frac{dx}{\cos\;\phi^{\prime }}\cos\;\phi^{\prime }=0,$$

where *σ_y_* represents the stress in the vertical direction. *σ_y_* = −*p* can be obtained because $\phi^{\prime }$ is very small. Considering the deformation as plane deformation, we can obtain *σ_x_* = 2*k* − *p*, where 2*k* = 1.155*σ_s_*. Taking into account the strain hardening in cold rolling, and neglecting the relatively small terms in the equation, Equation (13) can be rewritten as

(16) $$2ky\frac{d\left(\frac{p}{2k}\right)}{dx}\approx 2ktan\phi^{\prime }\pm p\mu ,$$

If *Y* = *p*/2*k*, then

(17) $$\frac{d\left(Y\right)}{dx}-\left(\pm \frac{\mu }{y}\right)Y\approx \frac{tan\phi^{\prime }}{y},$$

To sum up, Karman’s equation can be regarded as a special case when the roll speed in the force equilibrium differential equation is zero during Variable Thickness Rolling. It is worth noting that the force equilibrium equation is not continuous at the center line of the roll, so it may affect the calculation of force parameters such as the rolling force and even the precision of gap setting. Since the TRB has been produced stably, the above-mentioned problems may be ignored, or better mathematical equations may be constructed in the future.

#### 2.2.4. Horizontal and Vertical Velocity Field

The shape factor tan*ϕ*′ in the deformation area in the force equilibrium equation is the most important parameter in the equation. To determine its magnitude, a kinematic analysis of the VGR must be carried out. The shape of the transition area determines the relationship between the speed of the roll lifting and the actual horizontal rolling speed at the exit point of the rolling, while the shape of the deformation area determines the speed distribution of the workpiece throughout the deformation area. Only when the above relationships and speed distributions are made clear can the speed system be established and roll gap settings be made [38,40].

**Figure 6:**
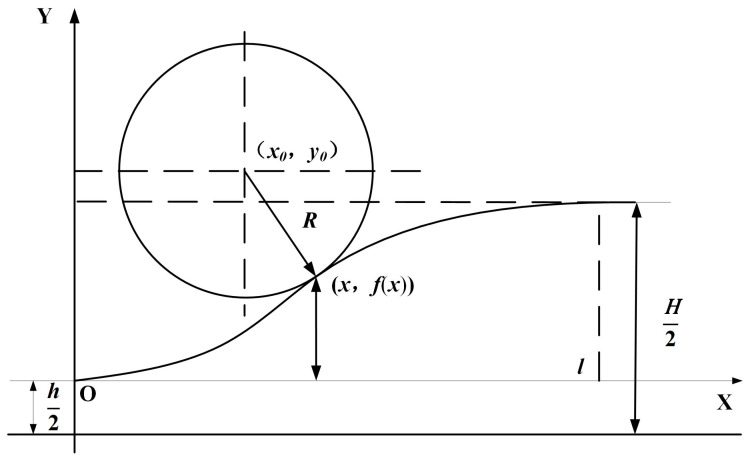
Schematic of the relationship between roll and workpiece [41].

The transition area curve is typically represented by a cubic function. For this type of transition area, Fang [41] derived the expression for the transition area curve from the thin area to the thick area.

(18) $$y=\frac{3}{2}\frac{H-h}{l^{2}}x^{2}-\frac{H-h}{l^{3}}x^{3},$$

where *H* is the thickness of the thick area, *h* is that of the thin area, and *l* is that of the transition area, as shown in Figure 6.

Based on this, considering that only when the vertical movement of the rolls matches the horizontal movement of the strip can the target transition area curve be achieved, the author derived the formula for the horizontal velocity at the exit and the vertical velocity of the rolls in the transition area rolling.

During the rolling of the transition area, the entire area is considered as the deformation area. Therefore, the metal undergoing deformation follows the principle of equal flow rate, which can be expressed as

(19) $$V_{x}\cdot h_{x}=C,$$

where *C* is the constant of the flow rate, *V_x_* is the rolling speed at the entrance of the thin area, and *h_x_* is the thickness of the thin area. At the point (*x*, *f*(*x*)),

(20) $$h_{x}=2\left[f\left(x\right)+\frac{h}{2}\right]=2f\left(x\right)+h,$$

Substituting Equation (18) into Equation (20) yields [41]

(21) $$V_{x}\left(x\right)=\frac{Cl^{3}}{\left(H-h\right)\cdot \left(3l-2x\right)\cdot x^{2}+hl^{3}},$$

Since the ratio of the vertical speed of the rolls at this point to the horizontal rolling speed is the slope, then

(22) $$V_{y}\left(x\right)=\frac{3C\cdot \left(H-h\right)\cdot \left(l-x\right)\cdot x}{\left(H-h\right)\cdot \left(3l-2x\right)\cdot x^{2}+hl^{3}},$$

Equation (21) represents the expression for the horizontal speed at the exit point during UR, and Equation (22) represents the expression for the lifting speed of the rolls. Under the condition of DR, the equation for the cubic curve in the transition area from the thick area to the thin area is as follows:

(23) $$y=\frac{3}{2}\frac{H-h}{l^{2}}{\left(l-x\right)}^{2}-\frac{H-h}{l^{3}}{\left(l-x\right)}^{3},$$

Similarly, the expression for the horizontal speed at the exit point during DR can be obtained:

(24) $$V_{x}\left(x\right)=\frac{Cl^{3}}{\left(H-h\right)\cdot \left(l+2x\right)\cdot {\left(l-x\right)}^{2}+hl^{3}},$$

The expression for the vertical speed of the rolls is

(25) $$V_{y}\left(x\right)=\frac{3C\cdot \left(H-h\right)\cdot \left(l-x\right)\cdot x}{\left(H-h\right)\cdot \left(l+2x\right)\cdot {\left(l-x\right)}^{2}+hl^{3}},$$

When the transition zone curve type is known to be a cubic curve, by using the above formula, the velocity of any point on the curve and the relationship between the horizontal velocity and the vertical velocity of the roll can be calculated; however, the unit pressure distribution in the transition zone can still not be obtained without the horizontal velocity distribution in the deformation zone.

**Figure 7:**
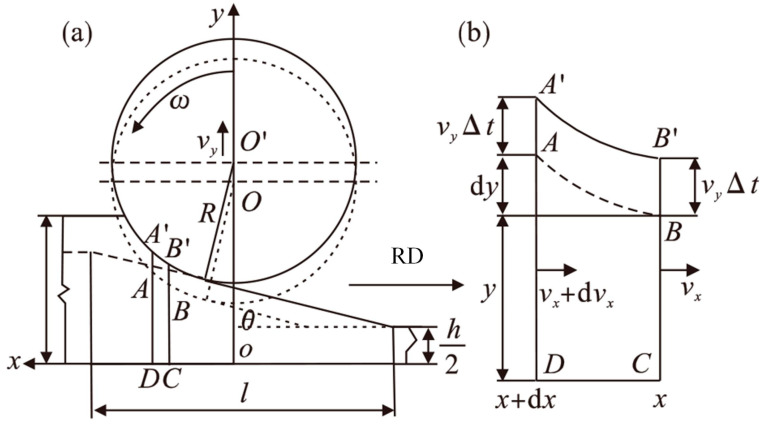
Schematic of UR process and element in deformation zone [43]. (**a**) Geometrical relationship of UR; (**b**) half element in deforming zone.

Based on the principle of volume invariance, Zhang [42,43] further studied the horizontal velocity distribution of metal in the deformation zone. The upward rolling is taken, as shown in Figure 7. At time *t*, the horizontal velocity of the micro element at BC (x) is *v_x_*, and at AD (*x* + d*x*) it is *v_x_* + d*v_x_*. Due to the lifting of the rolls, at time *t* + ∆*t*, the thickness of the workpiece at the positions *x* and *x* + d*x* changes from BC and AD to B′C and A′D, respectively. The horizontal velocity of the rolled piece during UR is a function of time *t* and position coordinate *x*. According to the condition of constant volume, it can be known that the metal flowing from the cross-section of the workpiece from the coordinate *x* + d*x* within time ∆*t* consists of two parts: one is the metal flowing out from the cross-section at the coordinate *x*, and the other is the metal enclosed by AA′B′B in Figure 7. It can be expressed as

(26) $$V_{x+dx}=V_{x}+V_{AA^{\prime }B^{\prime }B},$$

Assuming the vertical speed of the roll is *v_y_*, the volume of the workpiece flowing into the cross-section at *x* + d*x* within time ∆*t* can be obtained through integration:

(27) $$V_{x+dx}=\left(v_{x}+dv_{x}\right)\cdot ∆t\cdot \left(y+dy\right)+\frac{1}{2}\left(v_{x}+dv_{x}\right)\cdot v_{y}\cdot {\left(∆t\right)}^{2},$$

Similarly, the volume of the workpiece flowing out of the cross-section at *x* within time ∆*t* can be obtained:

(28) $$V_{x}=v_{x}\cdot ∆t\cdot y+\frac{1}{2}v_{x}\cdot v_{y}\cdot {\left(∆t\right)}^{2},$$

The volume enclosed by AA′BB′ can be expressed as

V*AA*′*B*′*B* = *vy*·∆*t*·d*x*, (29)

By combining Equations (26)–(29), we can obtain

(30) $$v_{x}dy+dv_{x}y+\frac{1}{2}v_{y}\cdot ∆t\cdot dv_{x}=v_{y}dx,$$

Let the contact arc function at time *t*_0_ be $y=f\left(x,t_{0}\right)$, substituting it into Equation (30) and simplifying, we can obtain

(31) $$\frac{dv_{x}}{dx}+\frac{f^{\prime }\left(x,t_{0}\right)}{f\left(x,t_{0}\right)+\frac{1}{2}v_{y}\cdot ∆t}v_{x}=\frac{v_{y}}{f\left(x,t_{0}\right)+\frac{1}{2}v_{y}\cdot ∆t},$$

The general solution of Equation (31) is [42]

(32) $$v_{x}=\frac{v_{y}x+C}{f\left(x,t_{0}\right)+\frac{1}{2}v_{y}\cdot ∆t},$$

Assuming that the UR starts at time 0, after a time of *t*_0_, the coordinate of the exit in the deformation area is *x* = *R*sin*θ*, the horizontal velocity of the exit of the workpiece is *v_x_* = *v_h_*_1_, and at the exit $y=f\left(Rsin\theta ,t_{0}\right)=h_{1}/2+v_{y}t_{0}$. *θ* is the inclination angle of the wedge section, *v_h_*_1_ is the actual horizontal velocity of the workpiece exit, which can be obtained through the speed roll at the exit, and *h*_1_ is the actual exit thickness at time 0. After the UR for time *t*_0_, the exit thickness satisfies

(33) $$\frac{h_{1}\left(t_{0}\right)}{2}=\frac{h_{1}}{2}+v_{y}t_{0}=\frac{h}{2}+R\left(1-\cos\;\theta \right)+v_{y}t_{0},$$

Substituting Equation (33) into Equation (32) and simplifying, we obtain

(34) $$C=\left(\frac{h_{1}}{2}+v_{y}t_{0}+\frac{1}{2}v_{y}∆t\right)\cdot v_{h1}-v_{y}\cdot R\sin\;\theta ,$$

Substituting Equation (34) into Equation (32), and letting ∆*t* approach 0, we obtain

(35) $$v_{x}=\frac{v_{y}\left(x-R\sin\;\theta \right)+v_{h1}\left(\frac{h_{1}}{2}+v_{y}t_{0}\right)}{f\left(x,t_{0}\right)}=\frac{v_{y}\left(x-R\sin\;\theta \right)+v_{h1}\frac{h_{1}\left(t_{0}\right)}{2}}{f\left(x,t_{0}\right)},$$

The analysis method of DR is the same as that of UR. By derivation, the horizontal speed of the workpiece during DR can be obtained as [42]

(36) $$v_{x}=\frac{-v_{y}\left(x+R\sin\;\theta \right)+v_{h1}\left[\frac{H}{2}-v_{y}t_{0}+R\left(1-\cos\;\theta \right)\right]}{f\left(x,t_{0}\right)}=\frac{-v_{y}\left(x+R\sin\;\theta \right)+v_{h1}\frac{h_{1}\left(t_{0}\right)}{2}}{f\left(x,t_{0}\right)},$$

Formulas (21) and (22), as the earliest velocity analytical formulas in the VGR process, lay a foundation for follow-up research and provide strong support for the industrial application of the VGR process. But they have their drawbacks. First, the calculation result is obtained based on the assumption that the equal discharge per second condition of each section in the deformation region holds. However, after analyzing Equation (35), it can be known that the second flow in the VGR process is a function of the coordinate *x*, showing that the second flow of each section is not equal at the same time. For UR, the second flow decreases gradually from the entrance to the exit. In addition, the research results of Fang [41] are based on the fact that the curve in the transition zone is a cubic curve. If the curve type changes, the relationship between horizontal and vertical velocities changes accordingly, resulting in the change in the final velocity formula. Thus, Equation (35) is a more reasonable and accurate velocity formula.

In addition, Zhang [44] discretized the VGR transition zone and simulated the rolling process. Based on the simulation results, the author determined the rolling speeds for the thick area and the thin area based on the rolling theory of the constant thickness area. They represented the rolling speed in the transition area simply as the average speed of the constant thickness area rolling.

(37) $$\bar{v}=\left(v_{h0}+v_{H0}\right)/2,$$

where $\bar{v}$ is the rolling speed in the transition area, $v_{h0}$ is that in the thin area, and $v_{H0}$ is that in the thick area. The rolling time in the transition area can be obtained using the following equation:

(38) $$t=l/\bar{v}=2l/\left(v_{h0}+v_{H0}\right),$$

where l is the length of the transition area. Since the vertical velocity in the transition area can be expressed as $v_{y}=\frac{\left(H-h\right)/2}{t}$, substituting Equations (37) and (38) into this expression gives [44]

(39) $$v_{y}=\frac{\left(H-h\right)\left(v_{h0}+v_{H0}\right)}{4l},$$

This derivation method is simple and practical, offering new ideas and theoretical guidance for the vertical motion control of rolls. But, due to significant simplifications and the improper simplification method of horizontal velocity, the calculation accuracy can be greatly affected. Although the validity of the mathematical model has been verified by finite element simulation, the lack of experimental verification makes the model less convincing. In addition, the author also over-looked a key point: the transition area shape was not considered.

**Figure 8:**
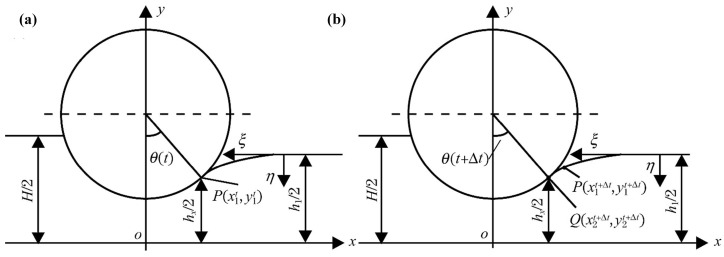
Schematic diagram for transitional zone′s rolling [45] (**a**) at time *t* and (**b**) at time *t* + ∆*t*.

The establishment of the above velocity field expression is based on the curve function often used in actual production. Wang et al. [45] put forward a double power function used for the transitional curve, which is continuous and smooth at all connection points, independent of its parameters, thus avoiding sudden changes in mechanical parameters during the rolling and forming process, as shown in Figure 8.

The authors established the horizontal velocity field of the rolled piece and the vertical velocity field of the roll suitable for this transitional curve [45]:

(40) $$v_{x}=\frac{v_{r}h_{\gamma }\cos\;\gamma +2Rv_{y}\left(\gamma +\theta \right)}{h_{x}},$$

where *γ* is the neutral angle; *v_r_* is the roller′s rotational velocity; and *h_γ_* is the workpiece′s thickness at the neutral point. *h_x_* is the workpiece′s thickness at the point of tangency.

(41) $$v_{y}=v_{r}\frac{h_{x}\cos\;\gamma +2R\left(\cos\;\theta -\cos\;\gamma \right)\cos\;\gamma }{h_{x}\cot\;\theta -2R\left(\gamma +\theta \right)},$$

The authors also pointed out the limitations of this velocity field expression, as it is not applicable to all transitional curve profiles. In order to ensure the validity of the formula, the local coordinate of the roll’s lowest point in the *η* direction must be less than half the difference between *h_1_* and *h_2_* until *ξ* = *L*. Here, *h*_1_ and *h*_2_ represent the thickness of the thick and thin areas, respectively, while *L* is the length of the transitional area.

At present, there are four different research results about the velocity in the deformation zone during the VGR process. It is obvious that the shape of the transition zone has a crucial effect on the velocity distribution, and different transition zone curves lead to the change in the matching relationship between the horizontal velocity of the workpiece and the vertical velocity of the rolls, thus causing the change in the velocity formula. The speed formulas of four research studies of transitional zone curves, such as cubic curve, straight line, and hyperbolic function, are established. Each model has its own characteristics and limitations, but Zhang’s [42,43] results are more realistic in that the characteristics and distribution of metal flow velocity in the deformation zone during VGR are described more accurately. Therefore, these results have also been successfully applied to industrial production.

#### 2.2.5. The Rolling Force Formula

At present, there are two types of rolling force models for the VGR process: one is a mathematical model based on the force equilibrium differential equation and the other is a rolling force model based on the energy method.

In references [30,39], the contact arc is regarded as a straight line, a common method in cold rolling. In the process of upwards cold rolling, the variation in strip thickness, denoted as 2*y*, through the roll gap can be expressed as *y* = *ax* + *b*, where the coefficients a and b can be calculated based on the thickness at the entrance and exit of the roll. Taking into account the influence of the inclination angle *θ*, the actual thickness at the exit is expressed as $h_{l}=h^{\prime }+2R\left(1-\cos\;\theta \right)$, Consequently, the value of y at the exit can be given by $y=h_{l}/2=h^{\prime }/2+R\left(1-\cos\;\theta \right)$.

Given that the deformation is characterized as plane strain and the material is incompressible, we can further obtain

(42) $$v_{h_{L}}\cdot v_{L}=v_{x}\left(x\right)\cdot 2y,$$

where $v_{x}\left(x\right)$ represents the horizontal velocity of the element at the x point, while $v_{h_{L}}$ and $v_{L}$ denote the velocity and thickness of the element at the actual exit.

To achieve a straight transitional zone, the equation $v_{y}=tan\phi \cdot v_{h_{L}}$ must be satisfied, where *v_y_* is the vertical velocity of the rolls. Therefore, Formula (17) can be expressed as [30]

(43) $$\frac{d\left(Y\right)}{dx}-\left(\pm \frac{\mu }{ax+b}\right)Y\approx \frac{ah_{L}-2tan\phi \left(ax+b\right)}{h_{L}\left(ax+b\right)},$$

The general solution to the above equation is

(44) $$Y=\frac{p}{2k}\approx C\cdot {\left(ax+b\right)}^{\pm \frac{\mu }{a}}-\frac{a}{\pm \mu }-\frac{2tan\phi }{h_{L}}\cdot \frac{1}{a-\left(\pm \mu \right)}\cdot \left(ax+b\right),$$

where *C* is a constant of integration.

Let *k*_1_ and *k*_2_ be the values of the yield shear stress at the entrance and exit, respectively. At the exit, $\sigma_{x}=q_{h_{L}}$, $q_{h_{L}}$ can be derived based on the actual forward tension stress in the thin area. Thus,

(45) $$p^{+}=2k_{2}-q_{h_{L}}=2\xi_{1}k_{2},$$

where $\xi_{1}=1-q_{h_{L}}/2k_{2}$. Also, at the entrance,

(46) $$p^{-}=2k_{1}-q_{H}=2\xi_{0}k_{1},$$

where $\xi_{0}=1-q_{H}/2k_{1}$ and *q_H_* is the back tension.

Combining Equations (44)–(46) and substituting *y* = *ax* + *b* = *H*/2 and *y* = *ax* + *b* = *h*/2 in Equation (44), respectively, produces the following:

For the backward sliding zone [30],

(47) $$\frac{p^{-}}{2k}\approx {\left(\frac{H}{2ax+b}\right)}^{\frac{\mu }{a}}\cdot \left(\xi_{0}-\frac{a}{\mu }+\frac{H}{h_{L}}\cdot \frac{tan\phi }{a+\mu }\right)+\frac{a}{\mu }-\frac{2tan\phi }{h_{L}}\cdot \frac{1}{a+\mu }\cdot \left(ax+b\right),$$

For the forward sliding zone,

(48) $$\frac{p^{+}}{2k}\approx {\left(\frac{2ax+2b}{h_{L}}\right)}^{\frac{\mu }{a}}\cdot \left(\xi_{1}+\frac{a}{\mu }+\frac{tan\phi }{a-\mu }\right)-\frac{a}{\mu }-\frac{2tan\phi }{h_{L}}\cdot \frac{1}{a-\mu }\cdot \left(ax+b\right),$$

where $a=\left[1/\left(l-R\sin\;\theta \right)\right]\left[\left(H-h^{\prime }\right)/2-R\left(1-\cos\;\theta \right)\right]$, $b=H/2-\left[l/\left(l-R\sin\;\theta \right)\right]\left[\left(H-h^{\prime }\right)/2-R\left(1-\cos\;\theta \right)\right]$, $x\in \left[R\sin\;\theta ,\;\;l\right]$. Through Equations (49) and (50), the distribution of unit pressure along the straight contact arc can be obtained under the condition of the straight transition area at any time during the upwards rolling.

When analyzing downwards rolling, the deformation zone should also be divided into Area I and Area II. The similar solution for Area I during downwards rolling can be written as follows:

For the backward sliding zone [30],

(49) $$\frac{p^{-}}{2k}\approx {\left(\frac{H}{2a_{I}x+b_{I}}\right)}^{\frac{\mu }{a_{I}}}\cdot \left(\xi_{0}-\frac{a_{I}}{\mu }-\frac{H}{h_{L}}\cdot \frac{tan\phi }{a_{I}+\mu }\right)+\frac{a_{I}}{\mu }-\frac{2tan\phi }{h_{L}}\cdot \frac{1}{a_{I}+\mu }\cdot \left(a_{I}x+b_{I}\right),$$

For the forward sliding zone,

(50) $$\frac{p^{+}}{2k}\approx {\left(\frac{2a_{I}x+2b_{I}}{h_{L}}\right)}^{\frac{\mu }{a_{I}}}\cdot \left({\xi_{1}}^{\prime }+\frac{a_{I}}{\mu }-\frac{tan\phi }{a_{I}-\mu }\cdot \frac{h^{\prime }}{h_{L}}\right)-\frac{a_{I}}{\mu }+\frac{2tan\phi }{h_{L}}\cdot \frac{1}{a_{I}-\mu }\cdot \left(a_{I}x+b_{I}\right),$$

where $a_{I}=\left(H-h^{\prime }\right)/2l$, $b_{I}=h^{\prime }/2$, $x\in \left[R\sin\;\theta ,l\right]$. ${\xi_{1}}^{\prime }=\xi_{1}\cdot exp\left(2\mu R\sin\;\theta /h^{\prime }\right)$, $x\in \left[0,l\right]$.

The solution for Area II during downwards rolling is given by

(51) $$\frac{p}{2k}=\xi_{1}\cdot exp\left[\frac{2\mu }{h^{\prime }}\left(x+R\sin\;\theta \right)\right],$$

where $x\in \left[-R\sin\;\theta ,0\right]$. Based on the above equations, we obtained the formula of the roll force as follows:

(52) $$P_{upwrads}=B\cdot \left(\int_{0}^{x_{n}}p^{+}dx+\int_{x_{n}}^{l}p^{-}dx\right),$$

(53) $$P_{downwrads}=B\cdot \left(\int_{x_{e}}^{0}pdx+\int_{0}^{x_{n}}p^{+}dx+\int_{x_{n}}^{l}p^{-}dx\right),$$

where *B* is the width of the blank, *x_n_* is the coordinate of the neutral plane, *l* is the length of the deforming zone, and *x_e_* is the coordinate of the exit in downwards rolling.

Another common treatment for the contact arc in cold rolling is to treat it as a parabola and then solve the force equilibrium equation [46]. There is no difference between the unit pressure and its subsequent solution, except that the contact arc equation is changed from a straight line to a parabola *y* = *ax*^2^ + *b*, so that Formula (17) can be expressed as

(54) $$\frac{d\left(Y\right)}{dx}-\left(\pm \frac{\mu }{ax^{2}+b}\right)Y\approx \frac{2a{x\cdot h}_{L}-2tan\phi \left(ax^{2}+b\right)}{h_{L}\left(ax^{2}+b\right)},$$

The general solution to the above equation is

(55) $$Y=\frac{p}{2k}\approx e^{\int \left(\pm \frac{\mu }{ax^{2}+b}\right)dx}\left[\int \frac{2a{x\cdot h}_{L}-2tan\phi \left(ax^{2}+b\right)}{h_{L}\left(ax^{2}+b\right)}e^{-\int \left(\pm \frac{\mu }{ax^{2}+b}\right)dx}dx+C\right],$$

The process of solving the integral constant and determining the boundary conditions to build the rolling force model is consistent with that mentioned above.

The first type of VGR force model is established in the same way as the conventional rolling force model, and there is no innovation in nature. When the force equilibrium differential equation in the deformation zone of VGR is established, different rolling force models are obtained by different contact arc treatments.

**Figure 9:**
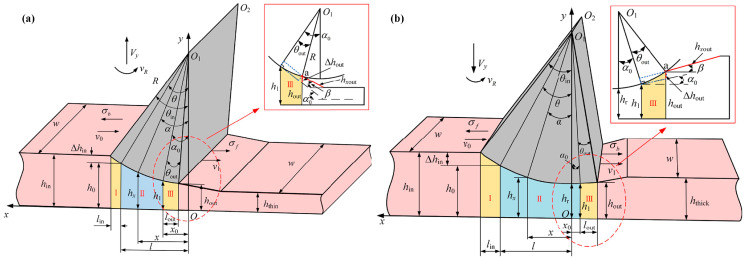
Three-dimensional diagram of deformation region during (**a**) UR and (**b**) DR. Here, region I is the entrance elastic compression region, II is the plastic deformation region, and III is the exit elastic recovery region [47].

Based on the principle of energy conservation, the second type of rolling force model solves the rolling force by calculating the deformation energy, friction energy and other energy changes during the VGR process. Liu [33] established a mathematical model for roll separating force in the TRB manufacturing process. Based on the minimum energy theory of the variational principle and considering the characteristics of the roll movement and workpiece deformation comprehensively, the internal plastic deformation, friction, shear and tension powers, and the minimum result of the total power functional in UR and DR are obtained. On this basis, Liu [47] further improved the method above. The elastic deformation of the strip and the flattening deformation of the roll are considered in order to improve the prediction accuracy of the results. The rolling deformation region is divided into elastic deformation and plastic deformation regions, as shown in Figure 9.

For the elastic deformation region, the roll separating force is calculated using the generalized Hooke’s law. In the plastic deformation region, the roll separating force is calculated using the energy method. By superimposing the roll separating force in the plastic and elastic deformation regions, the total roll separating force values that meet the convergence condition are acquired by using the coupling relationship between the force and the flattening radius.

Based on the above idea, the total rolling force during the upward rolling process is [47]

(56) $$F_{u}=F_{uin}^{e}+F_{uout}^{e}+F_{umin}^{p},$$

where $F_{uin}^{e}$ represents the roll separating force in the entrance elastic region I, $F_{uout}^{e}$ is the roll separating force in the exit elastic region III, and $F_{umin}^{p}$ is the roll separating force in the plastic region II. The specific formulas for these variables are provided in Appendix A.

Similarly, the total rolling force for the downward rolling process is [47]

(57) $$F_{d}=F_{din}^{e}+F_{dout}^{e}+F_{dmin}^{p},$$

where $F_{din}^{e}$ represents the roll separating force in the entrance elastic region I during DR, $F_{dout}^{e}$ is the roll separating force in the exit elastic region III during DR, and $F_{dmin}^{p}$ is the roll separating force in the plastic region II during DR. The specific formulas for these variables are also provided in Appendix A.

**Figure 10:**
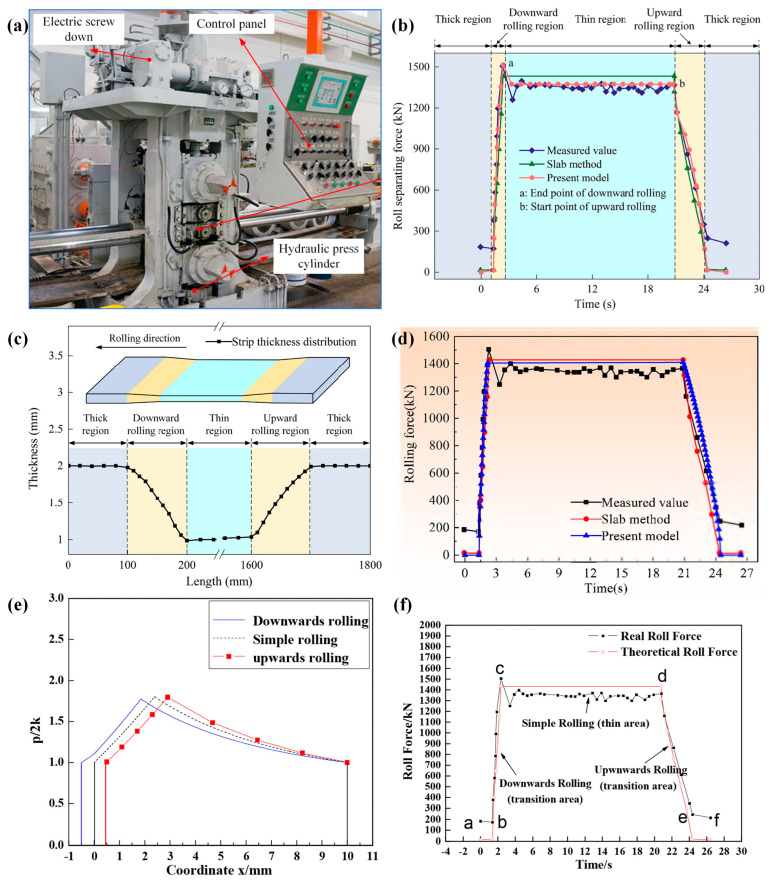
Experimental equipment of TRB rolling process and experimental results. (**a**) A 450 mm four-high cold-rolling mil; (**b**) the roll separating forces calculated with the present model are compared with the measured values and the slab method [47]; (**c**) dimensions of the strip after rolling; (**d**) the roll separating forces calculated with the present model are compared with the measured values and the slab method [33]; (**e**) comparison between simple rolling process and VGR process; (**f**) the roll separating forces calculated with the present model are compared with the measured values and the slab method [39].

Three mathematical models and experimental measurements of rolling force were compared, respectively. The results showed that the predictions of the three models are in good agreement with the actual values, as shown in Figure 10. The experiment used cold-rolled, high-strength, low-alloy-grade steel CR340. The specific material and process parameters are shown in Table 2.

With the same target workpiece size, TRBs with a final thickness difference ratio of 1:2 were achieved in the above three studies. The rolling force model established using the minimum energy method has better accuracy. The roll separating force calculated by the presentation model is larger than the calculated results using the slab method, and it is slightly larger than the measured data, as shown in Figure 10d. If the elastic deformation at the entrance and the exit and the flattening of the rolls are also considered on this basis, the accuracy of the obtained model will be further improved. The values calculated by the established model are basically consistent with the measured values, and both sets of data are slightly higher than the results obtained by the engineering method, as shown in Figure 10b. The rolling force model obtained by solving the conventional force balance equation also has good consistency with the measured values, and the error can be controlled within 10%, as shown in Figure 10f. In addition, the distribution of unit pressure along the contact arc under the following conditions—UR, DR, and CR—is also compared by using the conventional force balance equation, as shown in Figure 10e. It can be observed that the unit pressure in conventional rolling falls between that of UR and DR, which is a practical distribution characteristic. This is because the actual exit position of the deformation zone shifts to the left in UR, while it shifts to the right in DR.

Both the traditional method of constructing rolling force models and the use of the minimum energy principle to solve rolling force have been experimentally proven to be reasonable and accurate. Although the first method is based on plane deformation analysis, it is more widely used in engineering practice because it provides a quick means of estimating rolling force. The second method is more suitable for situations requiring high-precision calculations. Obviously, for VGR, the former method may be more appropriate. However, the latter method provides more accurate calculation results, which are also very meaningful and valuable for theoretical research and production guidance.

## 3. Technologies and Theories of BVT-TD

### 3.1. Introuctions of BVT-TD Technologies

A BVT-TD is produced by rolling, and it has a different thickness distribution along the transverse direction. The direction of thickness variation in this blank is exactly perpendicular to the direction of thickness variation in the BVT-RD. Although it is not too late to start technology development, compared with BVTs-RD, its research is still in its early stage. It is very difficult to produce blanks with variable thicknesses in the transverse direction that are also good quality for industrial applications. If a flat blank is rolled directly with rolls that have a variable thickness plate shape, serious plate shape problems or even fractures will occur due to the difference in deformation at different points. 

**Figure 11:**
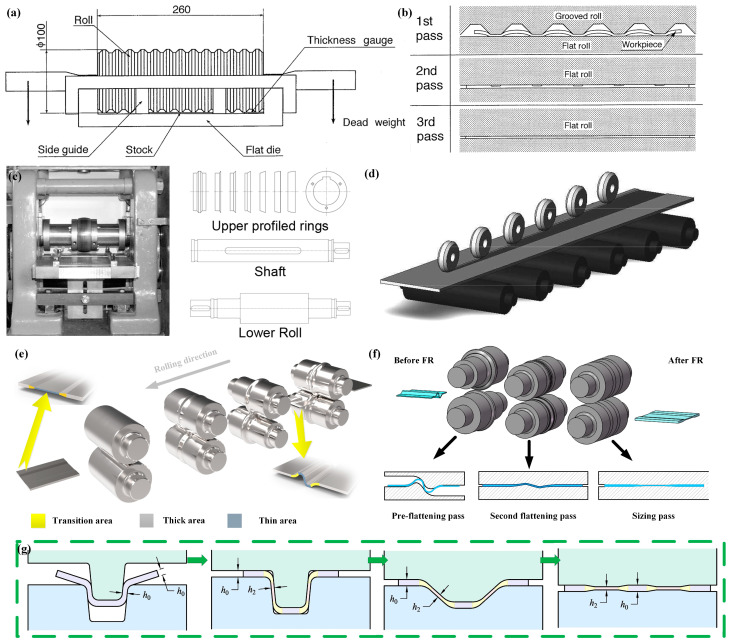
Technologies of BVT-TD. (**a**,**b**) Equipment and diagrammatic representation of the spread-rolling [48]; (**c**) roll structure of SPR [50]; (**d**) roll arrangement for SPR [51]; (**e**,**f**) BSR and FR processes of VTR [16]; (**g**) V-VTR process [21].

Utsunomiya [48] designed a rolling mill system with grooved rolls as the upper working rolls and flat rolls as the lower working rolls. With the action of these rolls, a part of the material is pressed down and another part protrudes into the grooves, and then the plate is gradually flattened with the flat rolls, thereby achieving lateral flow of the metal. Figure 11a,b show the process of Spreading–Rolling and the characteristics of its rolls. This was the earliest attempt to induce lateral metal flow by rolling. Taking a type of clay (plasticene) as the experimental material, the experiment consists of three passes. Due to the relatively small deformation zone, where the workpiece contacts the rolls, the rolling process can be approximated as strip rolling where the width spread cannot be ignored. This results in lateral metal flow. Although the experiment observed variations in thickness along the transverse direction, the difference in thickness was relatively small. Abo-Elkhier [49] made improvements in the size and quantity of the grooved roll mentioned above and conducted rolling experiments using aluminum as the experimental material. It was found that a strip 70 mm wide and 1 mm thick was widened by up to 3.1% for a reduction of 35%.

Although this method achieved lateral flow of the metal and an increase in the width of the sheet, the obtained thickness ratio difference was still very small, even when rolling materials with good plasticity and softness. In addition, this method had certain irrationalities. Some parts of the workpiece were deformed while other parts remained undeformed, which made the material more prone to issues such as shape defects or even fracture. After that, there were no further reports of related research, which also proved the low feasibility of this method. Although this method was not very successful, it provided an idea for achieving lateral flow of metal.

Kopp [50] proposed the Strip Profile Rolling (SPR) method to produce BVTs-TD, which are TRSs. This method envisions the use of a seven-roll system. The rolls consist of profiled rings which can be positioned on a shaft, as shown in Figure 11c, to produce a wide symmetrical groove in the middle of the strip during the rolling process. According to the width requirements of the thin zone, the number of rolls can be increased [52], as shown in Figure 11d [51]. This method can be seen as an extreme version of the approach proposed by Utsunomiya [48], which minimizes the width of profile rings and pass reduction. The rolling process is like a thin disk repeatedly cutting the blank, leaving a shallow groove on the blank after rolling. By changing the arrangement of rolls and using the same method on both sides of the groove, the groove size can be continuously widened. This method has achieved significant breakthroughs, obtaining a BVT-TD sample with a thickness ratio of approximately 1:1.7 using a laboratory rolling mill [53]. Despite this, this method also needs to address a series of shape issues caused by local deformation of the blank.

Another technique for producing BVTs-TD, which are RPSs, is Variable Thickness Rolling (VTR), which includes Bending–Spreading–Rolling (BSR) and Flattening Rolling (FR) [51]. BSR typically involves three to six rolling passes, where the uniformly thick flat workpiece is transformed into a curved workpiece with varying thicknesses through deformation, as shown in Figure 11e. Subsequently, FR, which involves three to five rolling passes, is used to flatten the curved workpiece. During the initial passes of the flattening rolling process, the workpiece does not undergo thickness changes, only shape alterations, with a minimal reduction occurring only in the final pass, resulting in a smooth and flat surface on the workpiece, as shown in Figure 11f. Metal flows both laterally and along the rolling direction (RD) with the special roll systems during the BSR process. This indicates that the thickness variation in the target area is caused by both rolling and stamping. Consequently, the metal in the thinned area elongates not only laterally but also along the RD, while the metal in the thickened areas flows only along the RD [16]. This transforms the strip, which initially has uniform thickness, into one with varying thickness distributions. This new method ensures that the elongation in the target thinned area is consistent with that in other areas, thus preventing the formation of waves and other shape defects resulting from non-uniform elongation in traditional flat rolling.

Building upon VTR, Wang et al. aimed to obtain BVTs-TD with a larger thickness difference ratio by modifying the roll system’s hole design and developed V-VTR with a V-shaped roll profile [21]. The entire V-VTR process consists of four passes: the V-shaped preforming pass, the V-shaped decreasing pass, the flattening pass, and the final pass, as depicted in Figure 11g. After V-VTR, RPSs with a thickness difference ratio of 1:2 were successfully obtained. This method shortens the rolling process of VTR, reduces the number of rolling passes, and achieves a larger thickness ratio. Additionally, the roll shape for this technique is simpler, with the roll profile curve mainly being straight lines, which improves the roll accuracy and reduces design difficulty.

### 3.2. Research Progress on These Processes

**Figure 12:**
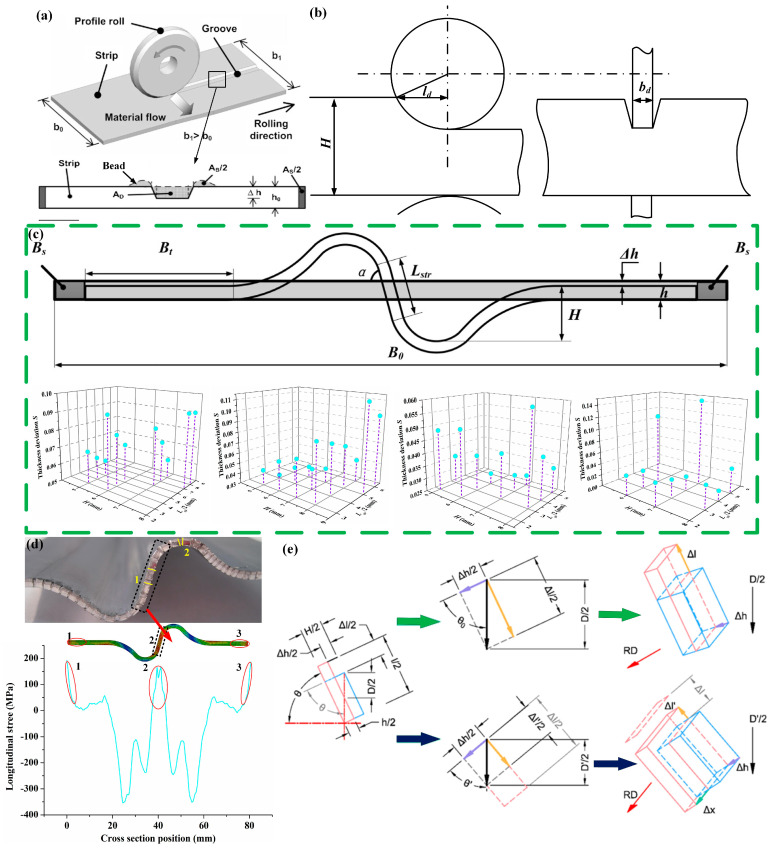
Research on BVT-TD. (**a**) Beads occurring in SPR [55]; (**b**) geometry meaning of contact width/contact length (*b_d_*/*l_d_*); (**c**) inflences of roll parameters in BSR [56]; (**d**) stress state in deformation zone of BSR [56]; (**e**) metal flows in different directions under different minimum critical angles [21].

In order to achieve thickness variation in the width direction, the metal can only flow laterally; any longitudinal flow would cause the strip to produce waves or small beads, as shown in Figure 12a. The geometry of the forming rolls and the contact area are the two variables that mainly influence the latitudinal material flow. The ratio of contact width (*b_d_*) to contact length (*l_d_*) in SPR has to be significantly small, as shown in Figure 12b [54]. Having conducted research through experiments and finite element simulations, Kopp [17] found that if the ratio is significantly smaller than 1.0, the lateral material flow dominates in the process, and can even achieve 100%. However, this requires very small pass reductions and a large number of rolling passes to ensure that the lateral compressive stresses beside the roll remain within the elastic range, thereby preventing bulging. Hirt [55] optimized the roll design and rolling sequence to produce a strip on a 12-stand roll forming mill manufactured by the company Dreistern. Beginning with a conventional strip made of DC01 steel (width 170 mm, thickness 2.5 mm), 29 rolling passes were necessary to achieve the desired geometry (width 186 mm, thickness 2.5 mm, with a longitudinal groove that is 64 mm wide where the thickness is reduced to 1.5 mm).

The roll profile is the key factor for VTR technology to successfully achieve the transverse thickness distribution of target size. Wang et al. [56] investigated the influence of roll parameters, including the obliquity of the curved deformation area (*α*), length of the straight part (*L_str_*), and the height of the curved deformation area (*H*) on the workpieces, as depicted in Figure 12c. The thickness deviation decreases as *α* increases, until it reaches 75°~80°. The thickness becomes more uniform and closer to the target value with the increase in *L_str_*.

The stress state and metal flow in the target thinned area were analyzed. The BSR alters the stress state of the deformation zone, which differs from traditional tension-free rolling with flat rolls. In target thinning areas, the stress of the workpiece is tensile in the RD and the stress state in the deformation zone changes from triaxial compression to uniaxial tension and biaxial compression, making it easier for the metal to yield and for thickness reduction, as depicted in Figure 12d. By changing the position of the roll profile on the roll body, different types of RPSs can also be obtained [57]. One BSR pass and four FR passes produce a type of RPS which is thick in the middle and thin on the sides, with a thickness difference ratio of 1:1.8, an increase compared to the thicker middle and thinner edges of RPSs. 

The study found that during the rolling process, the space provided by the rolls precisely accommodates the lateral metal flow. The presence of metal at the front and back ends of the workpiece’s cross-section obstructs its longitudinal flow. Under the opposing frictional forces on the upper and lower surfaces of the rolls, the metal predominantly undergoes lateral flow. The metal’s flow trajectory is indicated by the green arrows in Figure 12e. During the V-VTR roll design process, there is a critical angle. If the actual inclination angle $\theta^{\prime }$ during the rolling process is less than $\theta_{0}$, the lateral metal flow will be hindered, forcing longitudinal flow, as indicated by the purple arrows in Figure 12e. Therefore, this angle is crucial to the rolling process. It ensures that metal only flows laterally, thereby achieving a larger thickness variation ratio. Furthermore, the mathematical expression for this angle has been derived, and finite element simulations have been used to analyze the stress and strain states in the deformation zone at different minimum critical angles [21].

**Figure 13:**
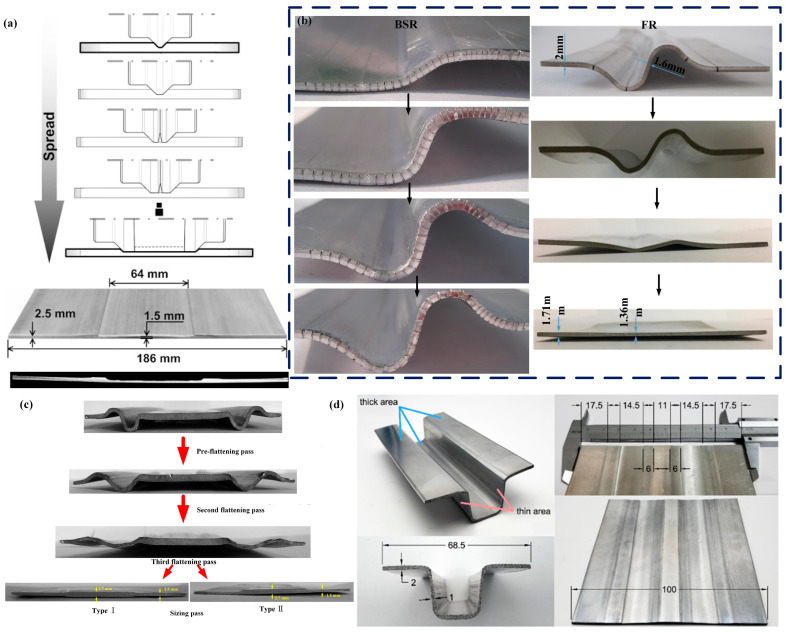
Different types of BVT-TD. (**a**) TRS [55]; (**b**) RPS which is thin in the middle and thick on the sides (by VTR) [16,56]; (**c**) RPS which is thick in the middle and thin on the sides (by VTR) [57]; (**d**) RPS (by V-VTR) [21].

Laboratory experiments have been conducted for all three BVT-TD production technologies, resulting in the production of BVTs-TD with different types and varying thickness variation ratios. Using a 12-stand roll forming mill and a total of 29 rolling passes, TRSs with a thickness variation ratio of 1:1.7 were successfully rolled, as shown in Figure 13a. By employing four BSR passes and three FR passes, a type of RPS which is thin in the middle and thick on the sides, with a thickness difference ratio of 1:1.3, was obtained, as shown in Figure 13b. By changing the roll profile position, another type of RPS that is thick in the middle and thin on the sides, with a thickness difference ratio of 1:1.8, was also successfully obtained. Additionally, by altering the final FR pass, symmetrical and non-symmetrical RPS types can be obtained based on demand, as shown in Figure 13c. After four passes of V-VTR, RPSs with a thickness variation ratio of 1:2 are shown in Figure 13d.

Each of the three BVT-TD technologies has its own characteristics. The SPR roll system is more flexible, with simpler processes and roll design. However, TRSs have limited thickness variation capabilities and are constrained by multiple rolling passes. Additionally, they can only achieve variable thickness on the upper surface of the blank, while the lower surface remains uniform. VTR can produce a wider range of RPSs, meeting the requirements for thickness distributions that are symmetric or asymmetric about the horizontal axis and offering greater flexibility in the location of thin areas in the product. The V-VTR process has the shortest technological process, which can significantly reduce equipment and production costs while also achieving a larger thickness variation ratio. This technology is more suitable for producing periodically variable thickness products and faces certain challenges in rolling RPS types that are thicker in the middle and thinner on the edges. Since SPR requires more rolling passes, it also means that more stands and supporting equipment are needed, so its production cost is higher than the other two processes. In addition, the workpiece produced by SPR only undergoes transverse metal flow, while the workpiece produced by the other two processes undergoes both longitudinal and transverse deformation. Therefore, the formability of TRSs may be better than that of RPSs. However, there is still no relevant research on the mechanical properties and formability of BVTs-TD.

### 3.3. Forming Theory of BVT-TD

Both of the theories of SPR and VTR are more complex compared to the common rolling theory. There is currently limited theoretical research on BVTs-TD, and extensive related work is urgently needed. Wang et al. [58] established a kinematically permissible velocity and strain fields based on the V-shaped roll deformation zone model and then derived the formulas for frictional work and strain energy. They used the upper bound theory to derive the rolling force and lateral force formulas for V-shaped rolling and experimentally validated the rolling force formulas.

#### 3.3.1. Velocity Field of Material in Deformation Zon

**Figure 14:**
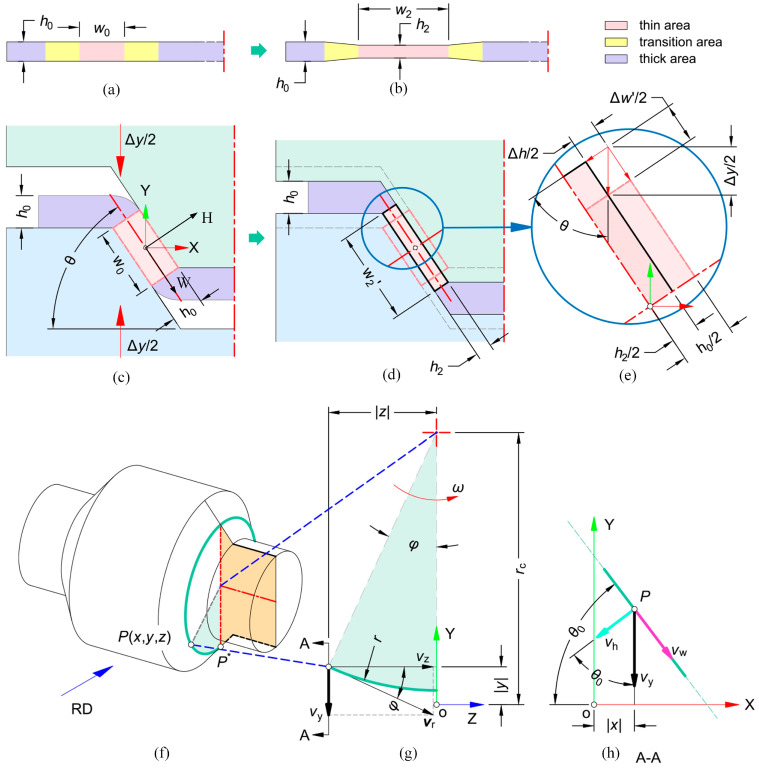
Dimensions of workpiece, deformation zone and geometry relationship of element in deformation zone of V-VTR. (**a**) The parameters of raw workpiece before V-VTR; (**b**) the parameters of target RPS in V-VTR; (**c**) the roll pass before thinning stage in V-VTR, where the roll moves down Δy during the thinning pass; (**d**) the roll pass after the thinning stage in V-VTR, where the thickness decreases to h_2_ and the width increases to w_2_′; (**e**) deformation of 1/4 thin area, where the Δy, Δh, and Δw are trigonometric; (**f**) the rotational trajectory of any point P on the surface of the rolling mill is a circular arc with a radius of r; (**g**) the components of the speed vector in *Y*-axis and *Z*-axis are v_y_ and v_z_, respectively; (**h**) the components of the speed vectors on XY-plane [58].

The deformation zone and the changes in the workpiece during V-VTR are illustrated in Figure 14. The velocity field at any point in the deformation zone can be derived based on the geometric relationships shown in Figure 14g,h. In the HWZ coordinate system, the velocity vector of point P can be represented as $v^{r}\left(v_{w}^{r},v_{h}^{r},v_{z}^{r}\right)$. Based on the geometric relationships shown in Figure 14g,h, we can obtain

(58) $$v_{h}^{r}=|\omega |\cdot z\cdot \cos\;\theta_{0},$$

where ω is the angular velocity and $\theta_{0}$ represents the critical angle. On the contact surface, relative tangential sliding occurs between the workpiece and the roll, while the relative velocity in the normal direction remains zero, indicating that $v_{h}$ is equal to $v_{h}^{r}$ on the contact surface. At *h* = *Hz*, $v_{h}$ is given by [58]

(59) $$v_{h}{=v}_{h}^{r}=|\omega |\cdot z\cdot \cos\;\theta_{0}=\frac{\left|\omega \right|z\sin\;\theta_{0}h}{H_{z}},$$

(60) $${\dot{\varepsilon }}_{h}=\frac{\left|\omega \right|z\sin\;\theta_{0}}{H_{z}},$$

where *H_z_* represents half of the thickness of the thin area.

According to the principle of volume conservation, ${\dot{\varepsilon }}_{w}+{\dot{\varepsilon }}_{h}+{\dot{\varepsilon }}_{z}=0$; thus, the strain rate in the transverse direction is

(61) $${\dot{\varepsilon }}_{w}=-{\dot{\varepsilon }}_{h}=-\frac{\left|\omega \right|\cdot z\cdot \sin\;\theta_{0}}{H_{z}},$$

Furthermore, the WZ-plane represents the symmetrical plane of the deformation zone within the WHZ coordinate system, implying that $v_{w}\left(0\right)=0$. The lateral component of the permissible kinematic velocity field denoted as $v_{w}$, is given by [58]

(62) $$v_{w}=\int {\dot{\varepsilon }}_{w}dw=-\frac{\left|\omega \right|\cdot z\cdot \cos\;\theta_{0}\cdot w}{H_{z}},$$

Furthermore, given that ${\dot{\varepsilon }}_{z}=0$, it is evident that the flow velocity of the metal in the RD remains constant. The velocity component $v_{z}$ in RD is not influenced by w, h, or z  [58].

(63) $$v_{z}=\left|\omega \right|\cdot r_{c},$$

where *r_c_* is the average roll radius. Based on the aforementioned expression for principal strain rate, the expression for shear strain rate can be derived as follows:

(64) $$\left\{\begin{array}{c} {\dot{\varepsilon }}_{wh}=\frac{1}{2}\left(\frac{\partial v_{w}}{\partial h}+\frac{\partial v_{h}}{\partial w}\right)=0\\ {\dot{\varepsilon }}_{wz}=\frac{1}{2}\left(\frac{\partial v_{w}}{\partial z}+\frac{\partial v_{z}}{\partial w}\right)=-\frac{\left|\omega \right|\cdot \cos\;\theta_{0}\cdot w}{2}\cdot \frac{2h_{2}}{{\left(\frac{h_{0}-h_{2}}{z_{1}}z+h_{2}\right)}^{2}}\\ {\dot{\varepsilon }}_{hz}=\frac{1}{2}\left(\frac{\partial v_{h}}{\partial z}+\frac{\partial v_{z}}{\partial h}\right)=\frac{\left|\omega \right|\cdot \cos\;\theta_{0}\cdot h}{2}\cdot \frac{2h_{2}}{{\left(\frac{h_{0}-h_{2}}{z_{1}}z+h_{2}\right)}^{2}} \end{array}\right.,$$

where *h*_0_ represents the thickness of the raw workpiece, *h*_2_ represents that of the thin area of RPS, and *z*_1_ connects the boundary point between deformation zones I and II. The division of the deformation zone is detailed in Appendix B.

#### 3.3.2. Rolling Force Formula

In V-VTR, the rolling force, denoted as $F_{R}$, consists of two components: the vertical component of the normal stress on the contact surface, $F_{hy}$, and the vertical component of the friction force on the same surface, $F_{fy}$:

(65) $$F_{R}=F_{hy}+F_{fy},$$

**Figure 15:**
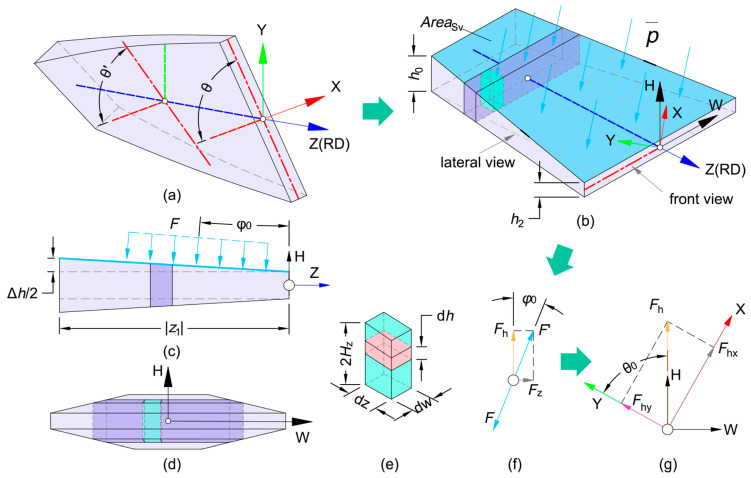
Simplification of deformation area and the forces used in the model [58]. (**a**) The ideal model of the deformation zone is a torsional body with a rectangular cross-section. (**b**) The simplified model of the deformation zone does not have torsion, and the new WHZ coordinate using width and thickness can describe the deformation zone size more easily. (**c**) A lateral view of the deformation zone, where the angle between the contact surface and *Z*-axis is *φ*_0_. (**d**) A front view of the deformation zone. (**e**) One element in deformation zone, the plastic strain energy, can be calculated by integrating this element. (**f**) The components of normal force in the *Y*-axis. (**g**) The components of normal force in the WH-plane.

In addition, $F_{hy}$ is the component of $F_{h}$ in the vertical direction. Given that $\varphi_{0}$ is negligible, $F_{h}\approx F^{\prime }=F$, and the entire deformation zone is subjected to the normal resultant force $F={Area}_{S_{v}}\cdot \bar{p}$, as shown in Figure 15. $\bar{p}$ is the maximum absolute value of the average pressure. The ${Area}_{S_{v}}$ is the area of the upper contact surface of the deformation zone.

According to upper bound theory [58],

(66) $$-\bar{p}\leq \frac{{\dot{W}}_{i}+{\dot{W}}_{f}}{2\left|\omega \right|\cos\;\theta_{0}{z_{1}}^{2}(\frac{w_{0}}{3}+\frac{w_{2}}{6})},$$

where ${\dot{W}}_{f}$ is the shear friction power and ${\dot{W}}_{i}$ is the internal plastic deformation power. *w*_0_ is the width of the thick area of the RPS before V-VTR and *w*_2_ is the width of the thick area of the RPS. The formulas of ${\dot{W}}_{f}$ and ${\dot{W}}_{i}$ are shown in Appendix C. Using the linear simplified model of the deformation zone, ${Area}_{S_{v}}$ is given by

(67) $${Area}_{S_{v}}=\frac{\left|z_{1}|(w_{0}+w_{2}\right)}{2},$$

Substituting Formulas (66) and (67) into $F={Area}_{S_{v}}\cdot \bar{p}$,

(68) $$F_{hy}=\cos\;\theta_{0}\cdot F_{h}=\frac{\left(w_{0}+w_{2}\right)}{4\left|\omega \right|\left|z_{1}\right|\left(\frac{w_{0}}{3}+\frac{w_{2}}{6}\right)}({\dot{W}}_{i}+{\dot{W}}_{f}),$$

Since only the component of the friction force along the W-axis that projects onto the *Y*-axis contributes to the rolling force, the component of the friction force in the Y-direction can be represented as [58]

(69) $$F_{fy}=\frac{m\sigma_{s}\sin\;\theta_{0}}{\sqrt{3}}\int_{z_{1}}^{0}\int_{{-W}_{z}}^{W_{z}}\frac{\frac{\left|\omega \right|z\cos\;\theta_{0}w}{H_{z}}-\left|\omega \right|z\sin\;\theta_{0}}{\left|∆v\right|}dwdz,$$

where *w_z_* is half of the width of the thin area.

## 4. Summary

A review of the technologies and theories regarding BVTs is conducted. According to the direction of thickness variation, BVTs can be divided into two typical products: BVTs-RD, with variable thicknesses along the rolling direction, and BVTs-TD, with variable thicknesses along the transverse direction. Due to their excellent strength and formability and reasonable load-bearing distribution characteristics, BVTs can be used as structural components. As a typical product of BVTs-RD, TRBs have already been widely used in car bodies, as underbody components, and in car crash boxes, etc., especially in the field of automotive manufacturing. Table 3 provides a summary of the technologies and theories regarding BVTs.

The technology of manufacturing BVTs-RD is Variable Gauge Rolling (VGR)—a type of flexible rolling. Through continuous adjustment to the roll gap, thickness variation along the RD is achieved. Compared with the manufacturing technology of Tailor Welded Blanks (TWBs), this method achieves better surface quality and weight reduction effects for the obtained product. In addition, this method is more efficient in production, resulting in a smooth transition of the product without stress peaks, thereby providing better formability. However, this method cannot be used for situations where materials with different thickness zones are different, and the flexibility of the thickness combination is slightly poor. Obviously, from the perspective of cost and performance, VGR is undoubtedly a better choice for producing BVTs-RD. The technologies of manufacturing BVTs-TD include Strip Profile Rolling (SPR) and Variable Thickness Rolling (VTR). The complete transverse metal flow is obtained during SPR by employing more than ten rolling mills with more than twenty passes. During VTR, both of the metal flows occur along the RD and TD, but the metal flow in the lateral direction is far more than that in the rolling direction. Although BVT-TD production technology has not yet achieved industrial applications, laboratory research results indicate that these technologies hold great promise for industrialization. In addition, if BVT-RD and BVT-TD production technologies are reasonably combined, a 3-D BVT with better weight reduction effects and lower costs can be obtained.

In addition, formulas for the biting angle, contact arc length, forward slip, rolling force, etc., of BVTs-RD are introduced. Different from the formulas for common rolling, which has unified formulas for rolling parameters, the formulas for BVT-RD rolling parameters usually have two expressions. One is for upward rolling, and the other is for downward rolling. A large amount of research work has been carried out on BVTs-RD, and the application of the technology is also well established. However, the technologies of BVTs-TD are still under development. Only the mathematical models of velocity field and the rolling force are studied. In addition, there are still no reports on industrial applications of or further theoretical research on BVTs-TD.

## 5. Conclusions

A BVT-RD production technology (VGR) and three BVT-TD production technologies (SPR, VTR, V-VTR) are introduced. A detailed review is provided on the characteristics and current status of theoretical research for these forming technologies. VGR has been maturely applied in industrial manufacturing, particularly in the automotive sector. However, BVT-TD manufacturing technology is still in its early stages of development, with significant room for growth in both theoretical and process research. Compared to TWB, workpieces produced through VGR offer advantages in weight reduction and formability, but the production process is more complex and the flexibility in material combinations is limited. BVT-TD production technologies cater to the varying load requirements along the width of the workpiece. SPR boasts a simple and flexible roll design but involves a longer process flow. While VTR and V-VTR enable a wider range of products, different specifications require matching roll combinations.

## Figures and Tables

**Table 1:** The biting angle and continuous rolling conditions.

Rolling Process	Bite Angle at Entrance [29,30]	Bite Angle at Entrance [31]	Bite Angle at Entrance [32]	*Φ* during the Rolling Process [29]	*Φ* during the Rolling Process [30]	Biting Condition	Continuous Rolling Condition
CR	$\alpha =\sqrt{∆h/R}$	$\alpha =\sqrt{∆h/R}$	$\alpha =\sqrt{∆h/R}$	$\frac{\alpha }{2}$	$\frac{\alpha }{K_{x}}$	*β* ≥ *α*	*β* ≥ *α* ≥ *φ*
UR	$\alpha =\sqrt{∆h/R}$	$\alpha =\sqrt{\frac{∆h}{R}}-\varphi$	$\alpha =\sqrt{H-h-2v_{y}t}$	$\frac{\alpha +\varphi }{2}$	$\frac{\alpha +(K_{x}-1)\varphi }{K_{x}}$	*β* ≥ *α*	*β* ≥ *α* ≥ *φ*
*DR*	$\alpha =\sqrt{∆h/R}$	$\alpha =\sqrt{\frac{∆h}{R}}+\varphi$	$\alpha =\sqrt{H-h+2v_{y}t}$	$\frac{\alpha -\varphi }{2}$	$\frac{\alpha +({1-K}_{x})\varphi }{K_{x}}$	*β ≥ α*	*β ≥ φ*

$K_{x}$ is the coefficient of the action point of the resultant force. *β* is the friction angle.

**Table 2:** Some rolling process parameters.

Parameter	Value [47]	Value [33,39]
Original strip thickness (mm)	2.2	2.2
Original width of strip (mm)	220	220
Forward tension of the strip (kN)	40	40
backward tension of the strip (kN)	40	40
Friction factor between the roll and the strip	0.075	-
Deformation resistance	$\sigma =323.3+631.08\varepsilon^{0.64544}$	$\sigma =323.3+32.3\varepsilon^{0.64544}$

**Table 3:** Summary of technologies and theories regarding BVTs.

Technologies	Type of Products	Technical Features			Technological Development Status	Theoretical Development Status
		Applicable Products and Cross-Sectional Shapes	Thickness Distribution Type	Pass Number		
VGR	BVT-RD	LP 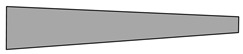	Wedge	/	Industrial application	Biting angle, contact arc length, forward slip, rolling force, etc.
		TRB 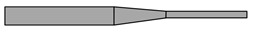	Wedge and periodic shape	/		
SPR	BVT-TD	TRS [55] 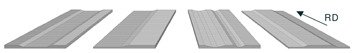	Asymmetrical shape	More than 20	Laboratory trial production	Rolling force
VTR		RPS [51] 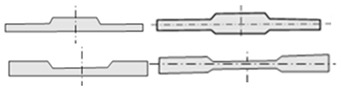	Symmetrical or asymmetrical shape	Less than 10		
V-VTR			Symmetrical or asymmetrical shape	4		

**Figure A1:**
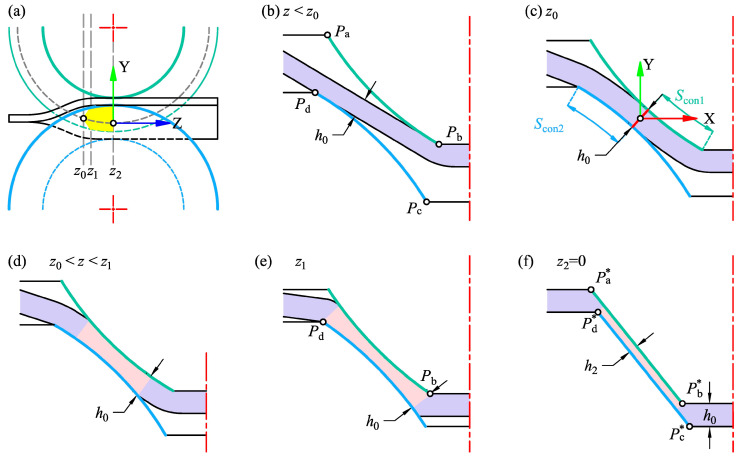
Ref. [58]. Variation in the deformation zone during the rolling process. (**a**) A lateral view of the rolls and workpiece; the contact surface of deformation zone is in yellow. (**b**) The section at any Z-plane *z* < *z*_0_; the workpiece is free and does not contact the roll face. (**c**) A section at *z* = *z*_0_; the value of the roll pass is equal to the thickness of the raw workpiece. (**d**) A section at any Z-plane *z*_0_ < *z* < *z*_1_; the thickness of the deformation zone is less than the thickness of the raw workpiece. (**e**) A section at *z* = *z*_1_; the contact surface touches the boundary points. (**f**) A section at *z*_2_ = 0; the thickness of the deformation is equal to the thickness of the thin area of the target Rolled Profile Strips.

## Appendix A

For the UR, in entrance elastic region I, the roll separating force $F_{uin}^{e}$ is

(A1) $$F_{uin}^{e}=\frac{2E_{s}wR}{\left(1-\upsilon_{s}^{2}\right)h_{in}}\left[\left(h_{in}-R-h_{r}\right)\left(\sin\;\theta_{in}-\sin\;\theta \right)+\frac{R}{2}\left(\theta_{in}-\theta +\frac{\sin\;2\theta_{in}-\sin\;2\theta }{2}\right)\right]\;+\frac{2\upsilon_{s}\sigma_{b}wR}{1-\upsilon_{s}}\left(\sin\;\theta_{in}-\sin\;\theta \right),$$

where σ_sin_ and σ_sout_ are the deformation resistances at the entrance and exit points of the strip plastic region, σ_f_ is the forward tension stress, and σ_b_ is the backward tension stress. vs. is Poisson’s ratio of the strip, and E_s_ is Young’s modulus.

In exit elastic region III, the roll separating force $F_{uout}^{e}$ is

(A2) $$F_{uout}^{e}=\frac{E_{s}wR}{\left(1-\upsilon_{s}^{2}\right)\left(h_{out}+h_{1}\right)}\left\{4\left[h_{out}-R-h_{r}-\left(x_{0}-l_{out}\right)\tan\;\beta \right]\left[\sin\;\alpha_{0}-\sin\left(\alpha_{0}-\theta_{out}\right)\right]\right.\;\left.+2R\tan\;\beta \left[{sin}^{2}\alpha_{0}-{sin}^{2}\left(\alpha_{0}-\theta_{out}\right)\right]+R\left[2\theta_{out}+\sin\;{2\alpha }_{0}-\sin\left({2\alpha }_{0}-{2\theta }_{out}\right)\right]\right\}\;+\frac{2\upsilon_{s}\sigma_{f}wR}{1-\upsilon_{s}}\left[\sin\;\alpha_{0}-\sin\left(\alpha_{0}-\theta_{out}\right)\right],$$

where $\theta =arcsin\left(l/R\right)$ and $l=\sqrt{2R\left(h_{0}-h_{r}\right)}$.

In plastic region II, the roll separating force $F_{umin}^{p}=M_{u}/\chi^{l}$, where Mu is expressed as follows:

(A3) $$M_{u}=\frac{R_{0}\left(\dot{W_{ui}}+\dot{W_{uf}}+\dot{W_{us}}\right)}{2v_{R}},$$

where $\dot{W_{ui}}$ is the internal plastic deformation power:

(A4) $$\dot{W_{ui}}=-\frac{16}{7}\sigma_{s}\left\{U\left[3-{\left(2+\frac{h_{1}}{h_{0}}\right)}^{e^{1-h_{1}/h_{0}}}\right]+2wV_{y}R\left(\theta -\alpha_{um}\right)\ln\;\left(\frac{h_{0}}{h_{1}}\right)+2wV_{y}R\left(\theta -\alpha_{0}\right)\right\},$$

where *α_n_* is a neutral angle. *U* is the plastic region’s entrance flow volume per second. *α_um_* is the average value of the angle corresponding to the plastic region during upward rolling.

$\dot{W_{us}}$ is the shear power of the plastic region:

(A5) $$\dot{W_{us}}=2kwh_{1}\tan\;\alpha_{0}\sqrt{{\left[\frac{U}{h_{0}w}e^{1-h_{1}/h_{0}}-\frac{V_{y}R\left(\theta -\alpha_{0}\right)}{h_{1}}-\frac{V_{y}R}{x_{0}}\right]}^{2}+{\left[\frac{U}{h_{0}}\left(\frac{1}{h_{0}}-\frac{1}{h_{1}}\right)e^{1-h_{1}/h_{0}}\right]}^{2}}\;+2kU\tan\;\theta -\frac{2kwh_{0}V_{y}R\tan\;\theta }{l},$$

$\dot{W_{uf}}$ is the friction power of the plastic region:

(A6) $$\dot{W_{uf}}=4mkw\left\langle v_{R}R\left(\theta +\alpha_{0}-2\alpha_{n}\right)+V_{y}R\ln\;\frac{{cos}^{2}\alpha_{n}}{\cos\;\theta \cos\;\alpha_{0}}+V_{y}R\left(\cos\;\alpha_{0}+\cos\;\theta -2\cos\;\alpha_{n}\right)\right.\;+\frac{2V_{y}R\left(\alpha_{um}-\theta \right)}{M\sqrt{M^{2}-1}}\left\{2arctan\left[\sqrt{\frac{M+1}{M-1}}\tan\left(\frac{\alpha_{n}}{2}\right)\right]-\right.arctan\left[\sqrt{\frac{M+1}{M-1}}\tan\;\left(\frac{\alpha_{0}}{2}\right)\right]\;\left.-arctan\left[\sqrt{\frac{M+1}{M-1}}\tan\left(\frac{\theta }{2}\right)\right]+\frac{\sqrt{M^{2}-1}}{2}\ln\;\frac{{tan}^{2}\left(\pi /4+\alpha_{n}/2\right)}{\tan\;\left(\pi /4+\alpha_{0}/2\right)\tan\left(\pi /4+\theta /2\right)}\right\}\;\left.+\frac{URg_{f}}{wh_{0}}\ln\;\left[\frac{\tan\left(\pi /4+\alpha_{n}/2\right)}{\tan\left(\pi /4+\alpha_{0}/2\right)}\right]+\frac{URg_{b}}{wh_{0}}\ln\;\left[\frac{\tan\left(\pi /4+\alpha_{n}/2\right)}{\tan\left(\pi /4+\theta /2\right)}\right]\;\right\rangle ,$$

$$h_{out}=h_{thin}+V_{y}t,$$

$$h_{r}=h_{out}+R\cos\left(\alpha_{0}-\theta_{out}\right)-R,$$

$$\alpha_{0}=arctan\left\{\frac{\left(h_{thick}-∆h_{in}\right)-\left(h_{thin}-∆h_{thinout}\right)}{L}\right\},$$

$$∆h_{in}=\frac{1-\upsilon_{s}^{2}}{E_{s}}h_{in}\left(\sigma_{sin}-\frac{\upsilon_{s}}{1-\upsilon_{s}}\sigma_{b}\right),$$

$$∆h_{out}=\frac{1-\upsilon_{s}^{2}}{E_{s}}h_{out}\left(\sigma_{sout}-\frac{\upsilon_{s}}{1-\upsilon_{s}}\sigma_{f}\right),$$

$$l_{in}=\sqrt{2R\left(h_{in}-h_{r}\right)-{\left(h_{in}-h_{r}\right)}^{2}}-\sqrt{2R\left(h_{0}-h_{r}\right)-{\left(h_{0}-h_{r}\right)}^{2}},$$

$$l_{out}=\sqrt{2R\left(h_{1}-h_{r}\right)-{\left(h_{1}-h_{r}\right)}^{2}}-\sqrt{2R\left(h_{out}-h_{r}\right)-{\left(h_{out}-h_{r}\right)}^{2}},$$

$$\theta_{in}=arcsin\left(\frac{l_{in}+l}{R}\right),$$

$$\theta_{out}=arcsin\sqrt{\frac{2R∆h_{out}\cos\;\alpha_{0}}{R}},$$

$$h_{1}=h_{r}+R-R\cos\;\alpha_{0},$$

$$∆h_{thinout}=\frac{1-\upsilon_{s}^{2}}{E_{s}}h_{thin}\left(\sigma_{sout}-\frac{\upsilon_{s}}{1-\upsilon_{s}}\sigma_{f}\right),$$

$$U=\upsilon_{0}wh_{0}=\upsilon_{R}\cos\;\alpha_{n}w\left(h_{0}+R\cos\;\theta -R\cos\;\alpha_{n}\right)+V_{y}Rw\left(\theta -\alpha_{n}\right),$$

$$\alpha_{um}=\frac{\int_{x_{0}}^{l}\alpha dx}{l-x_{0}}=\frac{\theta \sin\;\theta +\cos\;\theta -\alpha_{0}s\mathrm{in}\;\alpha_{0}-\cos\;\alpha_{0}}{\sin\;\theta -s\mathrm{in}\;\alpha_{0}},$$

$$g_{b}=e^{1-h_{mb}/h_{0}},$$

$$g_{f}=e^{1-h_{mf}/h_{0}},$$

$$h_{mb}=\frac{h_{\alpha_{n}}+h_{0}}{2},$$

$$h_{mf}=\frac{h_{\alpha_{n}}+h_{1}}{2},$$

$$M=\frac{h_{0}}{R}+\cos\;\theta ,$$

For the DR, in entrance elastic region I, the roll separating force $F_{din}^{e}$ is

(A7) $$F_{din}^{e}=\frac{2E_{s}wR}{\left(1-\upsilon_{s}^{2}\right)h_{in}}\left[\left(h_{in}-R-h_{r}\right)\left(\sin\;\theta_{in}-\sin\;\theta \right)+\frac{R}{2}\left(\theta_{in}-\theta +\frac{\sin\;2\theta_{in}-\sin\;2\theta }{2}\right)\right]\;+\frac{2\upsilon_{s}\sigma_{b}wR}{1-\upsilon_{s}}\left(\sin\;\theta_{in}-\sin\;\theta \right),$$

In exit elastic region III, the roll separating force $F_{dout}^{e}$ is

(A8) $$F_{dout}^{e}=\frac{-4E_{s}wR\sin\;\alpha_{0}}{\left(1-\upsilon_{s}^{2}\right)\left(h_{out}+h_{1}\right)}\left[\frac{R\tan\;\beta }{2}\sin\;\alpha_{0}+h_{out}-R-h_{r}-x_{0}\tan\;\beta \right]\;+\frac{4E_{s}wR\sin\left(\alpha_{0}+\theta_{out}\right)}{\left(1-{\upsilon_{s}}^{2}\right)\left(h_{out}+h_{1}\right)}\;\left.\left[\frac{R\tan\;\beta }{2}\sin\left(\alpha_{0}+\theta_{out}\right)+h_{out}-R-h_{r}-x_{0}\tan\;\beta \right]-\frac{E_{s}wR^{2}}{\left(1-\upsilon_{s}^{2}\right)\left(h_{out}+h_{1}\right)}\right\}\;\left[2\theta_{out}-\sin\;{2\alpha }_{0}+\sin\left({2\alpha }_{0}-{2\theta }_{out}\right)\right],$$

In plastic region II, the roll separating force $F_{dmin}^{p}=M_{d}/\chi^{l}$, where Mu is expressed as follows:

(A9) $$M_{d}=\frac{R_{0}\left(\dot{W_{di}}+\dot{W_{df}}+\dot{W_{ds}}\right)}{2v_{R}},$$

where $\dot{W_{di}}$ is the internal plastic deformation power:

(A10) $$\dot{W_{di}}=-\frac{16}{7}\sigma_{s}\left\{U\left[3-{\left(2+\frac{h_{1}}{h_{0}}\right)}^{e^{1-h_{1}/h_{0}}}\right]+2wV_{y}R\left(\theta -\alpha_{dm}\right)\ln\;\left(\frac{h_{0}}{h_{1}}\right)+2wV_{y}R\left(\theta -\alpha_{0}\right)\right\},$$

where α_dm_ is the average value of the angle corresponding to the plastic region during downward rolling.

$\dot{W_{ds}}$ is the shear power of the plastic region:

(A11) $$\dot{W_{ds}}=2kwh_{1}\tan\;\alpha_{0}\sqrt{{\left[\frac{U}{h_{0}w}e^{1-h_{1}/h_{0}}-\frac{V_{y}R\left(\theta +\alpha_{0}\right)}{h_{1}}+\frac{V_{y}R}{x_{0}}\right]}^{2}+{\left[\frac{U}{h_{0}}\left(\frac{1}{h_{0}}-\frac{1}{h_{1}}\right)e^{1-h_{1}/h_{0}}\right]}^{2}}\;+2kU\tan\;\theta -\frac{2kwh_{0}V_{y}R\tan\;\theta }{l},$$

$\dot{W_{df}}$ is the friction power of the plastic region:

(A12) $$\dot{W_{df}}=4mkw\left\langle v_{R}R\left(\theta -\frac{{sin2\alpha }_{0}}{2}-2\alpha_{n}\right)+V_{y}R\ln\;\frac{\cos\;\alpha_{0}{cos}^{2}\alpha_{n}}{\cos\;\theta }+V_{y}R\left(\cos\;\alpha_{0}+\cos\;\theta -2cos\alpha_{n}\right)\right.\;+\frac{2V_{y}R\left(\alpha_{dm}-\theta \right)}{M\sqrt{M^{2}-1}}\left\{2arctan\left[\sqrt{\frac{M+1}{M-1}}\tan\left(\frac{\alpha_{n}}{2}\right)\right]-\right.\left(2M^{2}-1\right)arctan\left[\sqrt{\frac{M+1}{M-1}}\tan\left(\frac{\alpha_{0}}{2}\right)\right]\;\left.-arctan\left[\sqrt{\frac{M+1}{M-1}}\tan\left(\frac{\theta }{2}\right)\right]+\frac{\sqrt{M^{2}-1}}{2}\ln\;\frac{{tan}^{2}\left(\pi /4+\alpha_{n}/2\right)\tan\left(\pi /4-\alpha_{0}/2\right)}{\tan\left(\pi /4+\theta /2\right)}-\alpha_{0}M\sqrt{M^{2}-1}\right\}\;\left.+\frac{URg_{f}}{wh_{0}}\left\{\ln\;\left[\tan\left(\pi /4+\alpha_{n}/2\right)\tan\left(\pi /4+\theta /2\right)\right]+2{\sin2\alpha }_{0}\right\}\;+\frac{URg_{b}}{wh_{0}}\ln\;\left[\frac{\tan\left(\pi /4+\alpha_{n}/2\right)}{\tan\left(\pi /4+\theta /2\right)}\right]\;\right\rangle ,$$

$$h_{out}=h_{thick}+V_{y}t,$$

$$h_{r}=h_{out}+R\cos\left(\alpha_{0}+\theta_{out}\right)-R,$$

$$\alpha_{0}=arctan\left\{\frac{\left(h_{thick}-∆h_{in}\right)-\left(h_{thin}-∆h_{thinout}\right)}{L}\right\},$$

$$∆h_{in}=\frac{1-\upsilon_{s}^{2}}{E_{s}}h_{in}\left(\sigma_{sin}-\frac{\upsilon_{s}}{1-\upsilon_{s}}\sigma_{b}\right),$$

$$∆h_{out}=\frac{1-\upsilon_{s}^{2}}{E_{s}}h_{out}\left(\sigma_{sout}-\frac{\upsilon_{s}}{1-\upsilon_{s}}\sigma_{f}\right),$$

$$l_{in}=\sqrt{2R\left(h_{in}-h_{r}\right)-{\left(h_{in}-h_{r}\right)}^{2}}-\sqrt{2R\left(h_{0}-h_{r}\right)-{\left(h_{0}-h_{r}\right)}^{2}},$$

$$l_{out}=\sqrt{2R\left(h_{out}-h_{r}\right)-{\left(h_{out}-h_{r}\right)}^{2}}-\sqrt{2R\left(h_{1}-h_{r}\right)-{\left(h_{1}-h_{r}\right)}^{2}},$$

$$\theta_{in}=arcsin\left(\frac{l_{in}+l}{R}\right),$$

$$\theta_{out}=arcsin\sqrt{\frac{2R∆h_{out}\cos\;\alpha_{0}}{R}},$$

$$h_{1}=h_{r}+R-R\cos\;\alpha_{0},$$

$$∆h_{thinout}=\frac{1-\upsilon_{s}^{2}}{E_{s}}h_{thin}\left(\sigma_{sout}-\frac{\upsilon_{s}}{1-\upsilon_{s}}\sigma_{f}\right),$$

$$U=\upsilon_{0}wh_{0}=\upsilon_{R}\cos\;\alpha_{n}w\left(h_{0}+R\cos\;\theta -R\cos\;\alpha_{n}\right)+V_{y}Rw\left(\theta -\alpha_{n}\right),$$

$$\alpha_{dm}=\frac{\int_{-x_{0}}^{l}\alpha dx}{l+x_{0}}=\frac{\theta \sin\;\theta +\cos\;\theta -\alpha_{0}s\mathrm{in}\;\alpha_{0}-\cos\;\alpha_{0}}{\sin\;\theta +s\mathrm{in}\;\alpha_{0}},$$

$$g_{b}=e^{1-h_{mb}/h_{0}},$$

$$g_{f}=e^{1-h_{mf}/h_{0}},$$

$$h_{mb}=\frac{h_{\alpha_{n}}+h_{0}}{2},$$

$$h_{mf}=\frac{h_{\alpha_{n}}+h_{1}}{2},$$

$$M=\frac{h_{0}}{R}+\cos\;\theta ,$$

## Appendix B

The division of the deformation zone during VTR is shown as follows:

Define deformation Zone I when *z*_0_ < *z* < *z*_1_ and deformation Zone II when *z*_1_ < *z* <0.

## Appendix C

The internal plastic deformation power ${\dot{W}}_{i}$ is given by

(A13) $${\dot{W}}_{i}=\frac{8\sqrt{3}}{3}|w|\cos\;\theta_{0}\sigma_{s}\int_{z_{1}}^{0}\int_{0}^{H_{z}}\int_{0}^{W_{z}}\sqrt{\frac{4z^{2}}{{\left(\frac{h_{0}-h_{2}}{z_{1}}z+h_{2}\right)}^{2}}+\frac{\left(w^{2}+h^{2}\right){h_{2}}^{2}}{{\left(\frac{h_{0}-h_{2}}{z_{1}}z+h_{2}\right)}^{4}}}dw\;dh\;dz,$$

The shear friction power ${\dot{W}}_{f}$ is given by

(A14) $${\dot{W}}_{f}=mk\left|\omega \right|\int_{z_{1}}^{0}\int_{-W_{z}}^{W_{z}}\sqrt{{\left(\frac{z\cdot \cos\;\theta_{0}\cdot w}{H_{z}}-z\cdot \sin\;\theta_{0}\right)}^{2}+{\left(\sqrt{{\left(r_{c}-a(w\cos\;\theta_{0}+x_{1})-b\right)}^{2}-z^{2}}-r_{c}\right)}^{2}}dw\;dz,$$
